# Rio Mamore Hantavirus Endemicity, Peruvian Amazon, 2020

**DOI:** 10.3201/eid3012.240249

**Published:** 2024-12

**Authors:** Marta Piche-Ovares, Maria Paquita García, Andres Moreira-Soto, Maribel Dana Figueroa-Romero, Nancy Susy Merino-Sarmiento, Adolfo Ismael Marcelo-Ñique, Edward Málaga-Trillo, Dora Esther Valencia Manosalva, Miladi Gatty-Nogueira, César Augusto Cabezas Sanchez, Jan Felix Drexler

**Affiliations:** Charité-Universitätsmedizin Berlin, Berlin, Germany (M. Piche-Ovares, A. Moreira-Soto, J.F. Drexler); Instituto Nacional de Salud, Lima, Peru (M. Paquita García, M.D. Figueroa-Romero, N.S. Merino-Sarmiento, A.I. Marcelo-Ñique, C.A. Cabezas Sanchez); Universidad Nacional, Heredia, Costa Rica (A. Moreira-Soto); Universidad Peruana Cayetano, Lima (E. Málaga-Trillo); Laboratorio de Referencia Regional de Salud Pública, Lambayeque, Peru (D.E. Valencia Manosalva); Laboratorio de Referencia Regional de Salud Pública, Loreto, Peru (M. Gatty-Nogueira); German Centre for Infection Research, Berlin, Germany (J.F. Drexler)

**Keywords:** Rio Mamore virus, hantaviruses, serology, genome, ecology, viruses, zoonoses, Peruvian Amazon, Peru

## Abstract

To explore hantavirus infection patterns in Latin America, we conducted molecular and serologic hantavirus investigations among 3,400 febrile patients from Peru during 2020–2021. Reverse transcription PCR indicated that a patient from Loreto, in the Peruvian Amazon, was positive for Rio Mamore hantavirus (serum, 3.8 × 10^3^ copies/mL). High genomic sequence identity of 87.0%–94.8% and phylogenetic common ancestry with a rodent-associated Rio Mamore hantavirus from Loreto in 1996 indicated endemicity. In 832 samples from Loreto, hantavirus incidence based on IgM ELISA of pooled Sin Nombre (SNV) and Andes virus (ANDV) nucleoproteins and immunofluorescence assay–based end-point titration using SNV/ANDV/Hantaan/Puumala/Saarema/Dobrava/Seoul hantaviruses was 0.5%. Across 3 ecologically distinct departments in Peru, SNV/ANDV IgG ELISA/IFA–based reactivity was 1.7%, suggesting circulation of antigenically distinct New World hantaviruses. Testing for arboviruses, nonendemic pathogens, and antigen-free ELISA corroborated nonspecific reactivity in 2 IgG and several IgM ELISA–positive serum samples. Hantavirus diagnostics and surveillance should be strengthened in Peru ad across Latin America.

Acute febrile illness (AFI) is a substantial public health problem in Latin America, exemplified by the almost US $3 billion expended annually for dengue outbreaks (*1*). The main cause of AFI in Latin America is dengue virus (DENV), followed by chikungunya virus, Zika virus, and *Plasmodium* spp. infections (*2*). However, ≈51% of AFI cases remain undiagnosed (*2*). The main reasons for the lack of elucidation of AFI etiology include similar clinical signs/symptoms and lack of robust and accessible diagnostic tools (*3*).

Hantavirus infections in the Americas can cause AFI and severe disease, termed hantavirus pulmonary syndrome (*4*). In South America, at least 12 human pathogenic hantaviruses cause hantavirus pulmonary syndrome, including Andes virus (ANDV), Laguna Negra virus (*5*), and Río Mamore virus (RIOMV). RIOMV has been documented from a single patient in Brazil in 2011, including only partial RIOMV genomic sequence characterization (*6*). RIOMV belongs to the species *Orthohantavirus mamorense,* which also encompasses Maripa and Laguna Negra viruses (family Hantaviridae, subfamily Mammantavirinae, genus *Orthohantavirus*). Human hantavirus infections are rodent-associated zoonoses (*7*). RIOMV in Peru was first described in 1996, isolated from a small-eared rice rat (*Oligoryzomys microtis*) from the department of Loreto in the Peruvian Amazon (*8*).

Hantavirus infection is infrequently diagnosed in humans because lack of testing and short viremia duration hinder direct detection (*7*). During 2011–2013, the Peruvian National Institute of Health (Instituto Nacional de Salud; INS) reported 6 human cases of hantavirus infection, all from Loreto (*7*). Through PCR-based detection and Sanger sequencing, 2 cases were determined by INS to be caused by the ubiquitous rat-associated Seoul virus and 2 by RIOMV; for 2 cases, the hantavirus could not be identified (*7*). However, the lack of viral genomic sequences in public databases and patient samples hinders confirmation of etiology. To learn more about the epidemiology, distribution, and risk factors of infection with RIOMV and other hantaviruses in Peru, we conducted a hantavirus-specific serologic and molecular investigation among persons with AFI who underwent medical investigation in Peru during 2020.

## Material and Methods

### Molecular Analyses

We extracted nucleic acids from samples collected during routine AFI surveillance in Peru by using the MagNA Pure 96 DNA and Viral NA Small Volume Kit (Roche, https://www.roche.com). To elucidate potential co-infections, we tested all samples by nested reverse transcription PCR (RT-PCR) for hantavirus RNA and samples from Loreto by quantitative RT-PCR (qRT-PCR) for DENV (*9*,*10*). We conducted library preparation by using the KAPA RNA HyperPrep Kit (Roche), followed by enrichment via in-solution hybridization capture (Arbor Biosciences, https://arborbiosci.com) as previously described for hantavirus genomic sequencing (*11*). We sequenced the captured library by using an Illumina Miniseq High-Output Reagent Kit (https://www.illumina.com) 150 cycles paired-end, and mapped reads against the RIOMV strain HTN-007 by using Geneious 2023.2.1 (https://www.geneious.com) and deposited sequence data in the European Nucleotide Archive (https://www.ebi.ac.uk; accession no. ERR11860590). We attempted to close sequence gaps by using PCR with specific bridging primers (Appendix 1 Table 1) and quantified the viral load by using a set of specific oligonucleotides (Appendix 1 Table 2).

### Phylogenetic Analyses

We conducted nucleotide alignments by using MAFFT with an L-INS-I algorithm (https://mafft.cbrc.jp) using Geneious 2023.2.1. We conducted Bayesian phylogenies by using MrBayes 3.2.6 (*12*) with a general time-reversible substitution model with gamma distribution and a complete deletion of all positions containing gaps in the alignment. We retrieved hantavirus reference sequences from GenBank for phylogenetic analysis (Appendix 1 Table 3). We conducted sequence identity plots of partial genomic sequences by using SSE 1.4 (*13*) with a fragment length of 200 and an increment between fragments of 50 nt. We constructed neighbor-joining trees of partial hantaviral genomic sequences in GenBank using 1,000 bootstrap replicates and the pairwise deletion option in MEGA X (*14*) (Appendix 1 Table 4). We determined translated amino acid sequence distances in MEGA X by using a pairwise deletion option (*14*).

### Serologic Analyses

We tested serum samples by IgM/IgG ELISA by using a pool of recombinant nucleoproteins from ANDV and Sin Nombre virus (SNV) licensed for diagnostic use in Peru (EUROIMMUN, https://www.euroimmun.com) (Appendix 1). To provide additional validation for the IgM/IgG ELISA-positive samples, we conducted an end-point antibody titration by using an indirect immunofluorescence assay (IFA), as previously reported for hantavirus serologic investigations (*15*,*16*). For IFA, we tested serum samples at 1:10–1:10,000 dilutions in 10-fold dilution steps. A few samples still yielded weak reactivity at 1:10,000, so we also tested those at 1:12,500. IFA was based on cells infected with ANDV, SNV, Seoul virus, Hantaan virus, Puumala (PUUV), Dobrava (DOBV), and Saaremaa hantaviruses (EUROIMMUN). For IgM-based IFA and ELISA, we pretreated serum samples with the immunoadsorbent Eurosorb (EUROIMMUN) to deplete class IgM rheumatoid factors potentially present in the sample that might react with specifically bound IgG, causing false-positive results and in parallel depleting specific IgG, displacing IgM from the antigen causing false-negative results. IgM and IgG detection relies on specific secondary fluorescein-coupled antibodies for IFA or horseradish peroxidase–coupled antibodies for ELISA.

To test for potential causes of unspecific reactivity in ELISA, we used PCR for pathogens commonly eliciting polyclonal B cell stimulation, including *Plasmodium* spp. (*17*), Epstein-Barr virus (EBV) (*18*), and cytomegalovirus (CMV) (*19*) (TIB Molbiol, https://www.tib-molbiol.de). To assess potentially unspecific reactivity, we used IgM/IgG ELISAs for endemic arboviruses in serum from hantavirus-seropositive patients and controls, including Oropouche virus (OROV) (*20*), Mayaro virus, chikungunya virus (*21*), and nonendemic arbovirus Crimean-Congo hemorrhagic fever virus (CCHFV), and we used IgG ELISAs only for *Plasmodium* spp. (*22*,*23*), SARS-CoV-2 (nucleoprotein antigen-based) (*24*), and the nonendemic pathogen Middle East respiratory syndrome coronavirus (S1-based; IgM is not commonly tested for respiratory human coronaviruses) (all ELISAs from EUROIMMUN).

To exclude a nonspecific reaction to components of the ELISA other than viral antigens, we tested all hantavirus IgM/IgG ELISA–positive samples on non–antigen-coated ELISA plates acquired from the manufacturer, following the same ELISA protocols. We compared serologic reactivity in hantavirus IgM/IgG ELISA-positive serum samples with 38 hantavirus IgM-negative and 38 IgG-negative samples from the same areas and time for which sufficient volumes were available, while ensuring comparable age distribution (hantavirus IgM-negative serum samples, mean patient age 20 years, SD 21.13; hantavirus IgM-positive serum samples, mean patient age 22 years, SD 22.58; *t*-test, *t* = 0.29, p = 0.77; hantavirus IgG-negative serum samples, mean patient age 26 years, SD 17.04; hantavirus IgG-positive serum samples, mean patient age 28 years, SD 24.53; *t-*test, *t* = 0.26, p = 0.80). We considered p<0.05 to indicate statistical significance and conducted all tests by using a 2-tailed approach. We performed statistical analyses by using R software version 2024.04.2 (The R Project for Statistical Computing, https://www.r-project.org).

### Virus Isolation

We used Vero E6 and BHK-21 cell lines for virus isolation attempts, as previously described for RIOMV and other hantaviruses (*25*). For both lines, we seeded a monolayer of 1.8 × 10^5^ cells per well in a 12-well dish with Dulbecco Eagle modified medium supplemented with 10% fetal bovine serum and 1% penicillin/streptomycin. We diluted the serum sample 1:25 and inoculated 250 μL onto the cell monolayer. After 1 hour, we removed the inoculum and replaced it with fresh medium. We checked the cells daily to assess the development of cytopathic effects and tested by qRT-PCR. We performed 2 blind passages over 1 week each.

## Results

### Cohort Description

**Figure 1:**
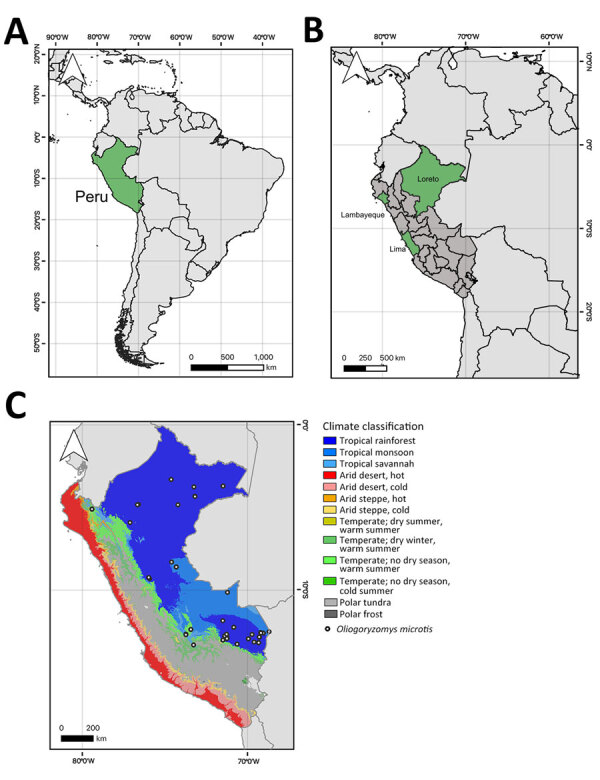
Locations and climate classifications related to study of Rio Mamore hantavirus endemicity in the Peruvian Amazon, 2020. A) Location of Peru (green) in South America. B) Regions in Peru where 3,400 serum samples from febrile patients were collected and stored during a dengue outbreak that overlapped with COVID-19 (*24*) in 3 diverse ecoregions: Loreto (Amazon; n = 1,972 samples), Lambayeque (coastal desert /dry forest; n = 743 samples), and Lima (coastal desert; n = 685 samples) (One Earth, https://www.oneearth.org). C) Climate classification regions of Peru and distribution of *Oligoryzomys microtis* small-eared rice rats (white dots) (https://www.gbif.org) (*26*). All maps were created by using QGIS 3.28.10 (https://hub.arcgis.com) based on freely available maps from Bucknell University.

During a 1-year period (January 2020–2021), INS collected and stored 3,400 serum samples from febrile patients for AFI surveillance during a DENV outbreak that overlapped with COVID-19 (*24*) in 3 diverse ecoregions: Loreto (Amazon; n = 1,972 samples), Lambayeque (coastal desert /dry forest; n = 743 samples), and Lima (coastal desert; n = 685 samples) (Figure 1). The mean age of patients analyzed was 27 years (SD 18.8); 51.1% (n = 1,735) were female and 48.9% (n = 1,663) male. The overall age and sex distributions were comparable to those of the total population of Peru (mean age 31 years; 50.8% female, 49.2% male) (https://www.inei.gob.pe/media/MenuRecursivo/publicaciones_digitales/Est/Lib1743/Libro.pdf).

### Molecular Testing for DENV and Hantaviruses

In January 2020, a serum sample from a 5-year-old boy from Iquitos, Loreto region, was positive for hantavirus RNA by nested RT-PCR (*9*) (Figure 1). Because sampling was conducted during a dengue outbreak and because other viruses may co-occur during dengue outbreaks, as was illustrated by detection of a Fort Sherman orthobunyavirus in a patient from Lambayeque (*27*), DENV infection in Loreto was assessed by qRT-PCR (*10*). The hantavirus-positive serum was negative for DENV, whereas the overall rate of DENV detection in Loreto was 56.8% (95% CI 54.7%–59.0%; n = 1,121/1,972) during 2020–2021. The patient experienced fever, muscle pain, headache, and vomiting for 3 days before sampling; no travel history preceding AFI was reported. No further information about the clinical signs/symptoms and disease outcome of the patient was available. A BLAST (http://blast.ncbi.nlm.nih.gov) comparison of the 347-bp region amplified by hantavirus RT-PCR after the exclusion of primer binding sites showed the highest nucleotide sequence identity (97.1%) to the rodent-associated RIOMV strain from 1996 in Loreto (*8*).

The viral load in the serum sample was low at 3.8 × 10^3^ copies/mL, which was consistent with the time since symptom onset, because the viral load is known to decline rapidly from ≈10^5^–10^6^ copies/mL 3–6 days after symptom onset (*28*). Virus isolation was unsuccessful, most likely because of low viral load and sample degradation under tropical conditions and repeated freeze–thaw cycles.

### Genomic Characterization of RIOMV

**Figure 2:**
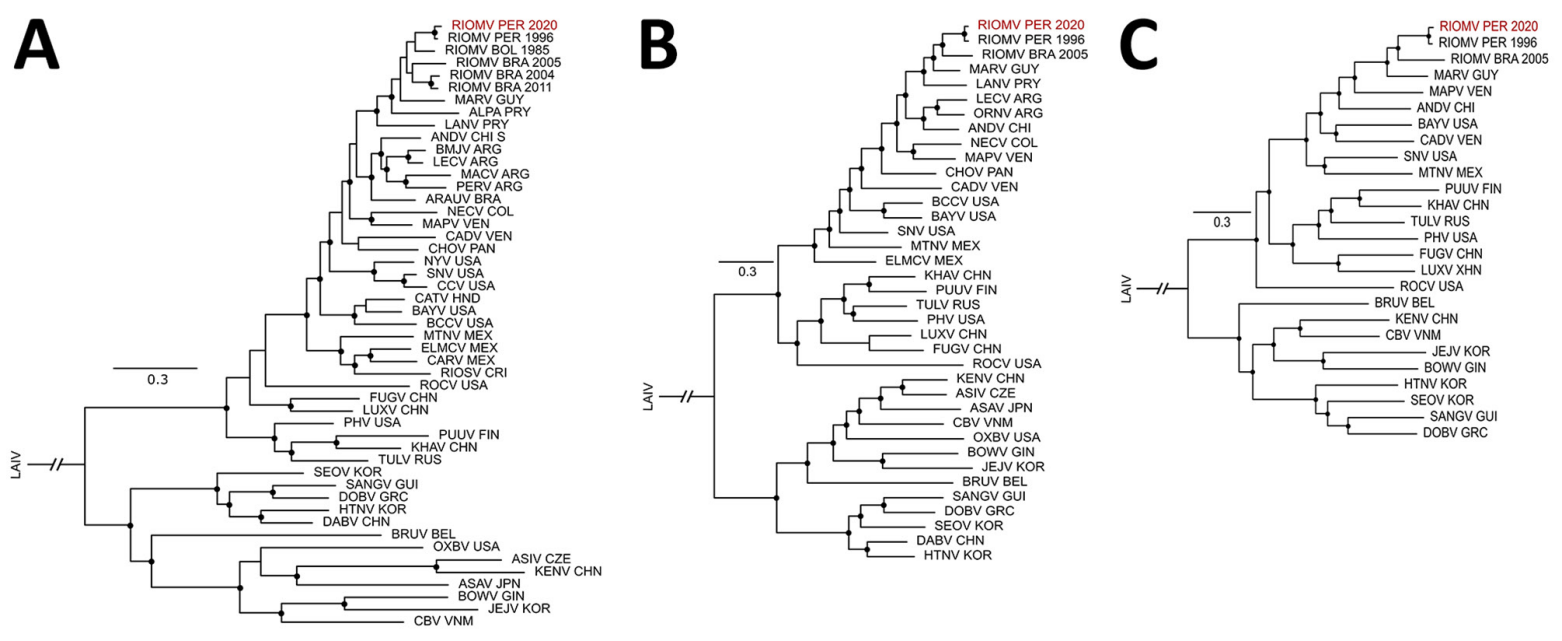
Phylogenetic relationships between partial concatenate sequences of RIOMV from Peru (RIOMV PER 2020, depicted in red) and reference sequences. The phylogenetic trees were constructed by using MrBayes 3.2.6 (http://mrbayes.csit.fsu.edu) and a general time-reversible substitution model with gamma distribution. Black circles at nodes indicate posterior probability >0.80. Reference sequences are available in Appendix 1. A) Partial sequence of the small segment (1,393 nt). B) Partial sequence of the medium segment (1,914 nt). C) Partial sequence of the large segment (1,617 nt). LAIN, Laibin mobatvirus

**Figure 3:**
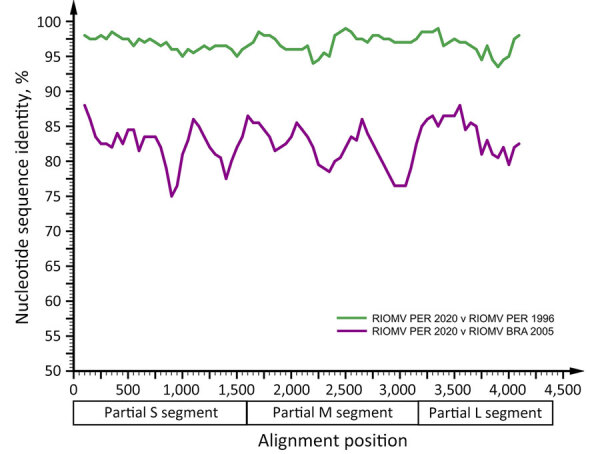
Sequence identity plot comparing RIOMV variants from Peru (RIOMV PER 2020 and RIOMV PER 1996) and Brazil (RIOMV BRA 2005). The identity plot was calculated by using SSE (http://www.virus-evolution.org) with partial concatenate sequences for the alignment of RIOMV (Appendix 1 Figure 2), a fragment length of 200 nt, and an increment between fragments of 50 nt. GenBank accession nos., RIOMV 1996 (FJ532244, FJ608550, FJ809772) and RIOMV Brazil 2005 (JX443679, JX443700, JX443697). L, large; M, medium; RIOMV, Rio Mamore virus; S, small.

Using a mixed approach of high-throughput and Sanger sequencing to close multiple gaps after high-throughput sequencing, probably because of low viral load, we reached a genome coverage of 41.4% (4,956 nt; 543,993 reads), of which 1,681 nt were from Sanger sequencing, with coverage of 72.7% for small (S), 52.5% for medium (M), and 24.7% for large (L) segments (GenBank accession nos. OR902838–40) (Appendix 1 Figure 1). Sequence comparisons within a dataset of all hantavirus reference sequences (Appendix 1 Table 3) showed that the 3 segments of the RIOMV strain from our study were most closely related to the rodent-associated RIOMV from 1996 (S segment, 96.8%; M segment, 96.4%; L segment, 97.0%). Separate Bayesian phylogenetic analyses of each segment placed the RIOMV strain consistently in a clade sharing the most recent common ancestry with the rodent-associated RIOMV (Figure 2). The phylogenetic placement of all genomic segments within the same RIOMV clade and similar sequence distance across genomic segments (Figure 3) spoke against reassortment or recombination events in the available dataset (*29*). The clustering of our sequence within the RIOMV clade was also supported by phylogenies relying on all available partial genomic sequences of viruses belonging to the *Orthohantavirus mamorense* species (Appendix 1 Table 4; Figure 2).

### Serologic Markers of Acute Hantavirus Infection

To investigate whether the molecular detection of RIOMV was epidemiologically linked to additional hantavirus cases, we selected all AFI samples from Loreto that were negative for DENV during January 2020–2021 (n = 832). We selected DENV-negative serum because acute DENV infection can affect serologic analysis for other pathogens (e.g., SARS-CoV-2) (*30*). Because a RIOMV-specific IgM ELISA was not available, we used a commercially available IgM ELISA licensed for diagnostic use in Peru that relies on pooled ANDV and SNV nucleoproteins, which are suitable for detecting hantavirus-specific immune responses shortly after infection (*31*). The IgM ELISA yielded a detection rate of 1.6% (95% CI 0.9%–2.7%; n = 13/832) averaged over 2020 (Appendix 2), including detectable IgM in the patient who tested positive by RT-PCR and exhibited high IgM IFA end-point titers of 1.0 × 10^4^ against SNV and ANDV individually (Table 1; Appendix 1 Figure 3, panel A). Among the 13 samples positive for IgM by ELISA, 30.8% (95% CI 12.4%–58.0%; n = 4/13) were positive by IgM IFA (Appendix 1 Figure 3, panel B). The incidence based solely on IFA results was 0.5% (95% CI 0.07%–1.11%; n = 4/832). All IgM-positive samples, except those positive by RT-PCR, were negative for IgG, which may be attributed to the facts that all patients were febrile and that IgG production is expected during the first weeks after symptom onset (Table 1) (*32*,*33*).

**Table 1:** Hantavirus IgM-positive serum samples from Loreto, Peru*

Sample	**IgM ELISA** **ratio†**	**Patient age, y/sex**	**Patient nationality**	**Date**	**Hantavirus** **RT-PCR**	**IgG ELISA ratio†**	**IFA result**
1395	**1.87**	1/F	Peruvian	2020 Jan	Negative	0.16	SNV-like 1.0 × 10^1^
3260	**5.00**	5/M	Peruvian	2020 Jan	Positive	**1.46**	SNV/ANDV-like 1.0 × 10^4^
3261	**4.00**	3/M	Peruvian	2020 Jan	Negative	0.15	Negative
3265	**1.36**	32/M	Peruvian	2020 Jan	Negative	0.17	Negative
3281	**1.22**	1/M	Peruvian	2020 Jan	Negative	0.57	Negative
3345	**2.96**	<1/M	Peruvian	2020 Jan	Negative	0.27	Negative
3358	**3.73**	45/M	Peruvian	2020 Sep	Negative	0.33	Negative
3376	**1.25**	27/M	Peruvian	2020 Sep	Negative	0.28	SNV-like 1.0 × 10^1^
3630	**2.60**	55/F	Peruvian	2020 May	Negative	0.17	Negative
4242	**2.60**	66/F	Peruvian	2020 Sep	Negative	0.28	SNV/ANDV/ PUUV-like 1.0 × 10^1^
4432	**2.03**	27/F	Peruvian	2021 Jan	Negative	0.33	Negative
4605	**1.11**	21/F	Foreign	2020 Dec	Negative	0.29	Negative
4893	**1.13**	2/M	Peruvian	2021 Jan	Negative	0.51	Negative

*ANDV, Andes virus; IFA, immunofluorescence assay; PUUV, Puumala virus; RT-PCR, reverse transcription PCR; SNV, Sin Nombre virus. †Results with a ratio greater than 1.1 are considered positive and marked in boldface.

**Figure 4:**
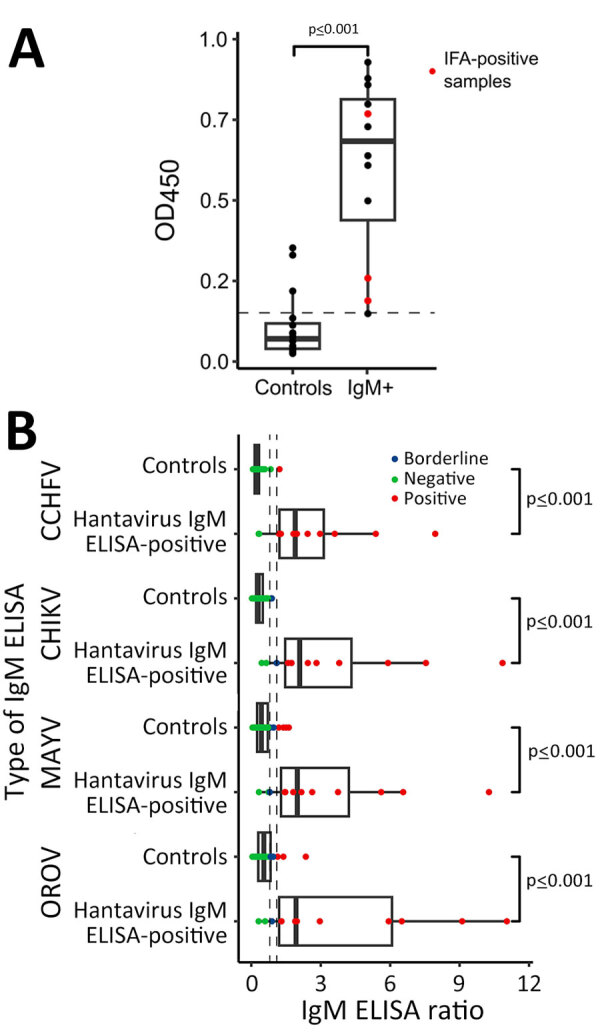
Investigation of unspecific reactivity in serum positive in hantavirus IgM ELISA, Peru. A) OD_450_ in noncoated ELISA plate. B) Comparison of IgM ELISA reactivity for different arboviruses: Control group, n = 38; hantavirus IgM–positive by ELISA group, n = 12. Tukey-style box plots are given with medians (thick lines within boxes) and interquartile ranges (box top and bottom or left and right edges); whiskers indicate 1.5× interquartile range. CCHFV, Crimean-Congo hemorrhagic fever virus, CHIKV, chikungunya virus, MAYV, Mayaro virus; OD_450_, optical density at 450 nm; OROV, Oropouche virus.

In January 2020, the 2 patients who were positive according to IgM IFA, including the patient who was positive according to RT-PCR, were 1 and 5 years of age. In September 2020, the patients who were IgM positive by IFA were a 27-year-old woman and a 66-year-old man. Beyond the patient positive by PCR, IgM end-point titers were generally low at 1.0 × 10^1^ against SNV and SNV/ANDV/PUUV, suggesting circulation of hantaviruses antigenically related to SNV/ANDV, potentially including RIOMV (Table 1; Appendix 1 Figure 4). Peru is not in the geographic distribution of the primary host of SNV, the North American deer mouse (*Peromyscus maniculatus*) (*34*), and PUUV is not endemic to South America. The reactivity for those viruses is probably explained by cross-reactivity between hantaviral epitopes, as previously described in full virus-based IFA (*35*).

In an IgM ELISA devoid of viral antigen, the optical density (OD) of hantavirus IgM IFA-confirmed serum did not differ significantly from that of IFA IgM-negative serum that had previously tested positive in the hantavirus IgM ELISA (Mann-Whitney *U* test, *U* = 20; p = 0.28) (Figure 4, panel A). We therefore compared reactivity with that of a control group composed of serum samples negative for hantavirus IgM by ELISA. The OD of serum samples showing reactivity in the hantavirus IgM ELISA was higher (mean 0.61, SD = 0.28) than that of serum samples nonreactive in the hantavirus IgM ELISA (mean 0.07, SD = 0.09; Figure 4, panel A). Among the IgM-positive samples, 83.33% (95% CI 54.0%–96.5%; n = 10/12) also showed reactivity in real-time PCR for CMV, EBV, and *Plasmodium* spp., and in IgM ELISAs for several endemic arboviruses (OROV, chikungunya, and Mayaro virus) and even for the nonendemic arbovirus CCHFV (Table 2). Arbovirus IgM ELISA reactivity in hantavirus IgM ELISA-reactive serum samples was consistently higher than that in hantavirus IgM ELISA-nonreactive serum samples (Figure 4, panel B). To avoid false-positive results, when calculating the IgM detection rate, we included only samples that tested positive for IFA.

**Table 2:** Results of complementary ELISA and PCR testing of hantavirus IgM serum samples positive by ELISA, Peru, January 2020–2021*

Sample	IgM ELISA ratio†					PCR copies/mL			Non–antigen coated plate OD
	CCHFV	CHIKV	MAYV	OROV		*Plasmodium* spp.	EBV	CMV	
**1395**‡	**1.19**	**1.59**	**1.46**	0.89		Negative	Negative	Negative	0.19
3261	**5.38**	**5.91**	**5.61**	**5.94**		Negative	Negative	Negative	0.80
3265	**1.82**	1.10	0.75	**1.30**		**8.4 × 10^5^**	**4.4 × 10^3^**	**1.9 × 10^3^**	0.93
3281	**1.97**	**2.82**	**3.75**	**6.50**		Negative	Negative	Negative	0.73
3345	**2.97**	**7.55**	**6.56**	**2.96**		Negative	Negative	**4.2 × 10^3^**	0.86
3358	**7.95**	**10.86**	**10.25**	**9.11**		**2.8 × 10^4^**	Negative	Negative	0.88
**3376**	0.34	0.65	0.80	0.60		Negative	Negative	Negative	0.26
3630	**3.61**	**3.80**	**2.17**	**1.30**		Negative	Negative	Negative	0.64
**4242**‡	**2.43**	**1.74**	**1.47**	**1.97**		Negative	Negative	Negative	0.77
4432	0.32	0.45	0.33	0.31		Negative	Negative	Negative	0.15
4605	**1.28**	**1.74**	**2.63**	**11.04**		Negative	Negative	Negative	0.50
4893	**1.22**	**2.44**	**1.81**	**1.90**		Negative	Negative	Negative	0.61

*Boldface indicates samples that tested positive. CCHFV, Crimean-Congo hemorrhagic fever virus; CHIKV, chikungunya virus; CMV, cytomegalovirus; EBV, Epstein-Barr virus; MAYV, Mayaro virus; OROV, Oropouche virus. The sample that tested positive by reverse transcription PCR was not tested because of limited sample volume after isolation attempts. OD of the negative control for IgM in Sin Nombre virus/Andes virus ELISA on a noncoated plate, 0.15. (Mann-Whitney *U* test, MAYV, *U *= 44; OROV, *U* = 56; CCHFV, *U* = 20, CHIKV, *U* = 15, p<0.001 for all). †Boldface indicates results with a ratio >1.1 considered positive in all ELISAs. ‡Samples that tested positive by hantavirus IFA.

### Serologic Markers of Past Hantavirus Infection

**Figure 5:**
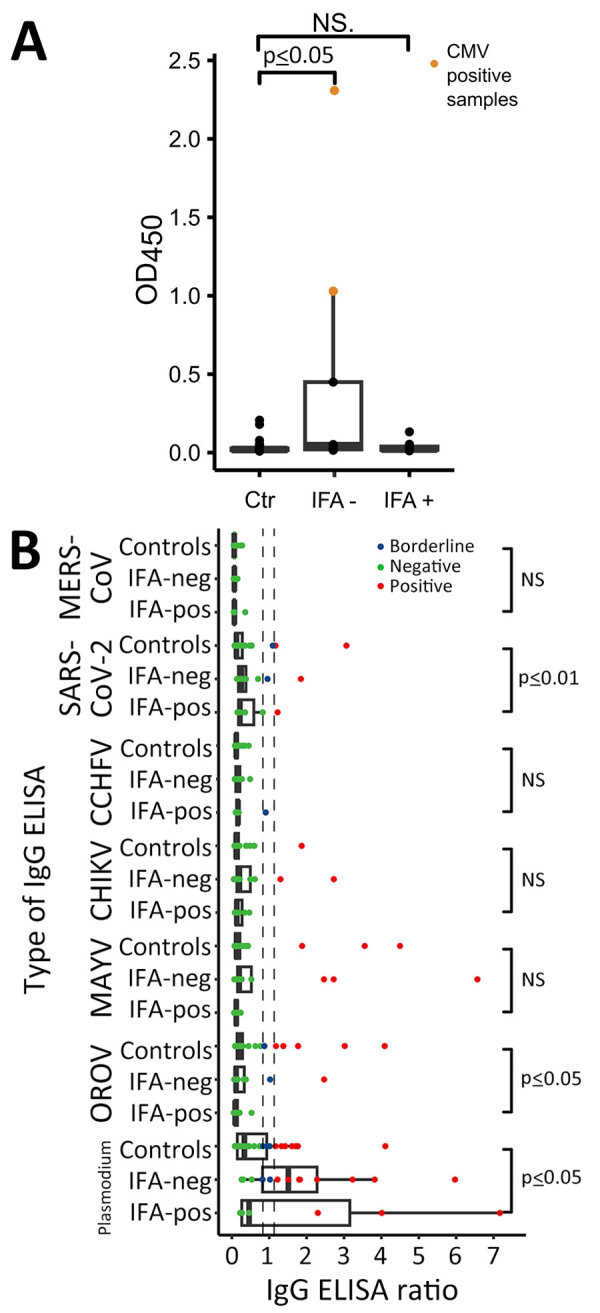
Investigation of unspecific reactivity in serum samples positive in hantavirus IgG ELISA, Peru. A) OD_450_ in noncoated ELISA plate. B) Comparison of IgG ELISA reactivity for different viruses excluding the CMV-positive samples. Control group, n = 38 ; hantavirus IgG IFA-negative samples, n = 7; hantavirus IgG IFA-positive samples, n = 13. Tukey-style box plots are given with medians (thick lines within boxes) and interquartile ranges (box top and bottom or left and right edges); whiskers indicate 1.5 × interquartile range. CCHFV, Crimean-Congo hemorrhagic fever virus, CHIKV, chikungunya virus; CMV, cytomegalovirus; IFA, immunofluorescence assay; MAYV, Mayaro virus; MERS-CoV, Middle East respiratory syndrome coronavirus; neg, negative; pos, positive; OD_450_, optical density at 450 nm; OROV, Oropouche virus.

The scarce hantavirus detections in humans and rodents in Peru all originated from the Amazon Basin. To compare whether persons living in other ecozones had had previous contact with hantaviruses, we tested 830 samples from Loreto, Lambayeque, and Lima that were DENV-negative by qRT-PCR and were of sufficient serum volume for hantavirus IgG testing by using the same ELISA that we used to detect IgM. The hantavirus IgG ELISA detection rate by region was 4.0% (95% CI 2.1%–7.0%; n = 11/278) in Loreto, 1.4% (95% CI 0.4%–3.8%; n = 4/279) in Lima, and 2.9% (95% CI 1.4%–5.8%; n = 8/273) in Lambayeque (Appendix 1 Figure 5, panel A; Appendix 2). We found no significant difference in hantavirus IgG detection rates by ELISA between sites (χ^2^ = 3.32; p = 0.20).

To confirm past hantavirus infections, we again performed IFA-based end-point titration. IgG IFA confirmed 60.9% (95% CI 40.7%–77.9%; n = 14/23) of the samples IgG ELISA-positive (Appendix 1 Figure 5, panel B). The sample positive by RT-PCR had IgG titers of 1.0 × 10^4^ against SNV and ANDV, a finding consistent with early IgG responses in some hantavirus-infected patients (*32*). End-point titration suggested 7 past infections with viruses antigenically more related to SNV than to ANDV, according to the highest SNV titers in all 3 ecozones. One serum sample each showed monotypic reactivity with SNV or ANDV (Table 3). Another 6 samples positive by IFA had similar titers against >2 hantaviruses, including DOBV/Saaremaa virus, Hantaan virus/DOBV, and SNV/ANDV (Table 3; Appendix 1 Figure 5, panel B). Nine samples positive by IgG ELISA samples were negative by IFA (Table 3).

**Table 3:** Hantavirus IgG-positive serum samples from Loreto, Lambayeque, and Lima, Peru, 2020*

Sample	Place	Patient age, y/sex	Nationality	Month	IgG ELISA ratio†	IFA result	Interpretation
1788	Lambayeque	13/M	Peruvian	Feb	**1.42**	SNV, 1.0 × 10^2^; ANDV/PUUV, 1.0 × 10^1^	SNV-like
1818	Lambayeque	6/M	Peruvian	Aug	**2.15**	Negative	Negative
1835	Lambayeque	29/F	Peruvian	Nov	**1.15**	Negative	Negative
1866	Lambayeque	60/M	Peruvian	Feb	**1.76**	Negative	Negative
1882	Lambayeque	20/F	Foreign	Mar	**2.24**	SNV 1.0 × 10^3^	SNV-like
1945	Lambayeque	4/F	Foreign	Jan	**1.52**	Negative	Negative
2738	Lambayeque	15/F	Peruvian	Mar	**1.35**	Negative	Negative
2740	Lambayeque	84/M	Peruvian	Mar	**1.77**	Negative	Negative
2003	Lima	48/F	Peruvian	Jan	**1.69**	SNV 1.0 × 10^1^	SNV-like
2069	Lima	19/M	Peruvian	Feb	**1.95**	SNV, 1.0 × 10^3^; ANDV, 1.0 × 10^2^; HTNV/SAAV/DOBV, 1.0 × 10^1^	SNV-like
2139	Lima	2/M	Foreign	Mar	**1.20**	Negative	Negative
2167	Lima	30/M	Peruvian	Jan	**2.80**	ANDV/SNV, 1.0 × 10^3^	ANDV/SNV-like
3136	Loreto	6/F	Peruvian	Jan	**1.64**	ANDV, 1.0 × 10^1^	ANDV-like
3249	Loreto	5/M	Peruvian	Jan	**1.16**	DOBV/SAAV, 1.0 × 10^1^	DOBV/SAAV-like
3260	Loreto	5/M	Peruvian	Jan	**1.46**	ANDV/SNV, 1.0 × 10^4^	ANDV/SNV-like
3317	Loreto	43/M	Peruvian	Jan	**1.17**	HTNV/DOBV, 1.0 × 10^1^	HTNV/DOBV-like
3361	Loreto	22/M	Peruvian	Sep	**2.31**	ANDV/SNV, 1.0 × 10^2^	ANDV/SNV-like
3404	Loreto	16/M	Peruvian	Sep	**1.12**	SNV, 2.5 × 10^3^; ANDV, 1.0 × 10^2^; PUUV, 1.0 × 10^1^	SNV-like
3562	Loreto	<1/F	Peruvian	Mar	**1.96**	Negative	Negative
3615	Loreto	16/M	Peruvian	Apr	**1.67**	SNV, 1.0 × 10^3^; ANDV/HTNV/DOBV, 1.0 × 10^1^	SNV-like
3937	Loreto	<1/M	Peruvian	Mar	**5.30**	Negative	Negative
4524	Loreto	26/F	Peruvian	Dec	**3.16**	ANDV/SNV, 1.0 × 10^4^; HTNV/PUUV/SEOV/DOBV, 1.0 × 10^1^	ANDV/SNV-like
4591	Loreto	71/M	Peruvian	Jan	**1.69**	SNV 2.5 × 10^3^; ANDV 1.0 × 10^2^; PUUV 1.0 × 10^1^	SNV-like

*Boldface indicates positive results (ratio >1.1). ANDV, Andes virus; DOBV, Dobrava virus; HTNV, Hantaan virus; IFA, immunofluorescence assay; PUUV, Puumala virus; SAAV, Saaremaa virus; SEOV, Seoul virus; SNV, Sin Nombre virus.

Complementary testing as before revealed that 2 serum samples (Table 4, samples 3562 and 3937) from children <1 year of age that were positive by the hantavirus IgG ELISA but negative by hantavirus IgG IFA were CMV positive by PCR. In an IgG ELISA plate devoid of viral antigens, the ODs in the 2 CMV-positive serum samples were strikingly higher at 1.03 and 2.31 (mean 1.67, SD = 0.9) compared with the rest of the samples that were IgG ELISA-positive samples (mean 0.05, SD = 0.09). The ODs of samples that were positive by hantavirus IgG ELISA but negative by hantavirus IgG IFA,, including those 2 samples, differed significantly from those of the control group (Table 4; Figure 5, panel A). After excluding the 2 CMV-positive samples, we detected no significant differences between the ODs of the 3 compared groups. The 2 CMV-positive samples also showed significantly higher reactivity in most complementary ELISAs, including pathogens not found in South America, such as CCHFV and Middle East respiratory syndrome coronavirus (Table 4). After excluding the 2 CMV-positive samples, we found significant differences in ELISA reactivity levels for OROV, SARS-CoV-2, and *Plasmodium* spp. (Figure 5, panel B), whereas the overall number of positive serum samples did not differ among groups (Appendix 1 Table 8). Thus, we calculated the ELISA-based IgG detection rate without the 2 CMV-positive serum samples. The adjusted hantavirus IgG detection rate for Loreto was 3.2% (95% CI 1.6%–6.1%; n = 9/278;); 66.7% (95% CI 45.2%–82.9%; n = 14/21) of the samples also tested positive by IFA, and the overall IgG detection rate was 1.7% (95% CI 1.0%–2.8%; n = 14/830) across the 3 ecozones.

**Table 4:** Results for complementary ELISA and PCR testing of hantavirus IgG-positive serum samples by ELISA serum samples, Peru, January 2020–2021*

Sample	IgG ELISA ratio†								PCR copies/mL			Non–antigen coated plate/OD
	CCHFV	CHIKV	MAYV	OROV	MERS-CoV	SARS-CoV-2	*Plasmodium* spp.		*Plasmodium* spp.	EBV	CMV	
**1788**	0.12	0.11	0.15	0.05	0.06	0.15	2.25		Negative	Negative	Negative	0.04
1818	0.07	0.09	0.03	0.02	0.02	0.79	0.23		Negative	**1.2 × 10^3^**	Negative	0.03
1835	0.11	0.31	0.04	0.06	0.03	0.18	0.19		Negative	Negative	Negative	0.02
1866	0.15	0.06	0.07	0.06	0.02	0.12	**3.98**		Negative	Negative	Negative	0.02
**1882**	0.05	0.07	0.12	0.04	0.03	0.11	**5.94**		Negative	Negative	Negative	0.01
1945	0.87	0.44	0.21	0.50	0.33	0.32	**7.14**		Negative	Negative	Negative	0.45
**2003**	0.06	0.03	0.03	0.04	0.02	0.11	**3.79**		Negative	Negative	Negative	0.01
**2069**	0.13	0.02	0.07	0.03	0.02	0.15	**1.48**		Negative	Negative	Negative	0.01
2139	0.13	0.10	0.09	0.18	0.04	0.17	0.24		Negative	Negative	Negative	0.05
**2167**	0.17	0.17	0.21	0.34	0.08	0.23	**1.77**		Negative	Negative	Negative	0.05
2216	0.10	0.06	0.06	0.06	0.03	0.17	0.37		Negative	Negative	Negative	0.01
2738	0.08	0.05	0.13	0.06	0.04	0.14	**2.27**		Negative	Negative	Negative	0.02
2740	0.15	0.17	0.09	0.10	0.04	**1.19**	0.43		Negative	Negative	Negative	0.05
**3136**	0.24	0.18	0.08	0.07	0.12	0.22	0.23		Negative	Negative	Negative	0.13
**3249**	0.21	0.17	0.16	0.13	0.04	0.20	0.50		Negative	Negative	Negative	0.04
**3317**	0.08	0.58	**2.43**	0.99	0.02	0.34	0.79		Negative	Negative	Negative	0.02
**3361**	0.06	0.10	0.17	0.08	0.03	0.92	**3.20**		Negative	Negative	Negative	0.02
**3404**	0.08	0.12	0.23	0.06	0.05	0.13	0.99		Negative	Negative	Negative	0.03
3562	**2.71**	**2.02**	**2.30**	**2.97**	0.71	**1.66**	**2.78**		Negative	Negative	**1.7 × 10^3^**	**1.03**
**3615**	0.08	0.46	0.49	0.09	0.03	0.30	0.27		Negative	Negative	Negative	0.02
3937	**5.48**	**3.89**	**2.71**	**3.69**	**2.05**	**3.56**	**3.45**		Negative	Negative	**2.1 × 10^3^**	**2.31**
**4524**	0.18	**2.70**	**6.54**	0.29	0.04	0.67	**1.79**		Negative	Negative	Negative	0.03
**4591**	0.46	**1.27**	**2.69**	**2.44**	ND	**1.82**	**1.19**		Negative	Negative	Negative	0.02

*Boldface indicates samples that tested positive by hantavirus IFA (ratio >1.1). The RT-PCR positive sample was not tested because of to limited sample volume after isolation attempts. CCHFV, Crimean-Congo hemorrhagic fever virus; CHIKV, chikungunya virus; CMV, cytomegalovirus; EBV, Epstein-Barr virus; IFA, immunofluorescence assay; MAYV, Mayaro virus; MERS-CoV, Middle East respiratory syndrome coronavirus; ND, not done; OD, optical density; OROV, Oropouche virus. Samples that tested positive by hantavirus IFA indicated in bold. †OD of negative control for IgG in Sin Nombre virus/Andes virus ELISA on a noncoated plate, 0.01. Statistical results comparing the ratios of hantavirus IFA-positive, IFA-negative serum samples, and the control group, Kruskal-Wallis; CCHFV, *H* = 2.33, p = 0.31; CHIKV, *H* = 2.0, p = 0.22; MAYV, *H* = 5.0, p = 0.08; MERS-CoV, *H* = 0.01, p = 0.99; OROV, *H* = 8.24, p = 0.02; *Plasmodium* spp., *H* = 12.21, p = 0.02 and SARS-CoV-2, *H* = 10.12, p = 0.006.

## Discussion

We detected and characterized RIOMV and analyzed hantavirus exposure for patients with AFI in Peru. Our serologic results were consistent with those of a previous study conducted in Loreto during 2007–2010 that used an ANDV antigen–based capture ELISA and reported an IgM detection rate of 0.3% (n = 15/5,174) (*36*). Moreover, the high genomic similarity of the RIOMV strain from our study and the rodent-associated RIOMV from 1996 and the geographic distribution of the host in the Amazon suggest endemicity (*37*) (Global Biodiversity Information Facility, https://www.gbif.org). That the only human case described from Brazil also occurred in the Amazon Basin reaffirms the area of RIOMV endemicity and confirms the ability of RIOMV to cause disease in humans (*6*).

The ecology of hantavirus infections is complex and probably varies according to climatic factors, predator/prey relationships, land use changes, host abundance, and virus genetics (*38*). Most cases of infection with ANDV, which is hosted by rodent species of a different genus than those of the genus that hosts RIOMV (*34*), in Argentina and Chile have been reported during spring and summer, when food availability is higher (*39*). In the Amazon region, the relatively constant climatic conditions throughout the year and the continuous harvest of different crops make it challenging to identify risk factors (*40*). However, 2 samples confirmed IgM positive were collected in January and another 2 were collected in September 2020, suggesting the potential for seasonal patterns of hantavirus infection in Loreto.

The lower sensitivity of IFA compared with ELISA may also partly explain the observed difference between ELISA and IFA reactivity (*41*,*42*), especially when recombinant nucleoproteins are used rather than full virus-infected cells as antigens (*43*,*44*) and when RIOMV antigens are not included. Moreover, the nonspecific reactivity in the hantavirus ELISA is compatible with polyclonal B cell activation resulting from CMV, EBV, *Plasmodium* spp., or hantavirus infections (*45*) and emphasizes the value of confirming IgM/IgG ELISA results, ideally by neutralization tests (NTs) for IgG (*15*,*16*).

Ecologic investigations of hantaviruses in the Peruvian Amazon and their host are thus urgently needed. Further investigation of circulating hantavirus strains in humans in Peru and other regions of South America is also warranted because our serologic findings suggest that antigenically diverse hantaviruses may co-occur.

Among the potential limitations of our study is the use of uneven numbers of samples throughout the year. It is likely that, during the onset of the COVID-19 pandemic, movement restrictions and lack of medical personnel led efforts to be focused on COVID-19, and persons with febrile illness without respiratory signs/symptoms might not have sought medical care (*46*,*47*). That interpretation is consistent with lower numbers of reported cases of dengue during the onset of the COVID-19 pandemic in Latin America (*47*). In addition, virus NTs might have contributed additional serologic information. However, the cross-reactivity of hantavirus immune responses would probably also have limited unambiguous results in virus NTs, even if we had an RIOMV isolate (*44*). In addition, a comparison of glycoprotein-based IFA- and NT-based serotyping results in a previous study of Old World hantaviruses was consistent in 79.5% of the samples (*42*). Thus, our conclusions, which are based on exhaustive serologic testing, are most likely robust even in the absence of NTs.

The early signs/symptoms of hantavirus infection are nonspecific (*6*,*7*,*48*) and resemble those of other AFIs. Therefore, it is necessary to enhance AFI surveillance by incorporating diagnostic protocols for hantavirus in patients in Peru and other ecologically similar regions of Latin America with compatible symptomatology and no evidence of dengue or malaria.

Appendix 1 Additional information for study of Rio Mamore hantavirus endemicity, Peruvian Amazon, 2020.

Appendix 2 Supplementary information for study of Rio Mamore hantavirus endemicity, Peruvian Amazon, 2020.
