# Supplementary material for: Rio Mamore Hantavirus Endemicity, Peruvian Amazon, 2020
Source: Emerg Infect Dis. 2024 Dec;30(12):2532–43. doi: 10.3201/eid3012.240249 (PMC11616641; doi:10.3201/eid3012.240249)
Supplement: Appendix 1 — Additional information for study of Rio Mamore hantavirus endemicity, Peruvian Amazon, 2020. [file 24-0249-Techapp-s1.pdf]

Article DOI: https://doi.org/10.3201/eid3012.240249

EID cannot ensure accessibility for supplementary materials supplied by authors. Readers who have difficulty accessing supplementary content should contact the authors for assistance.

Rio Mamore Hantavirus Endemicity, Peruvian Amazon, 2020

Appendix 1

ELISA procedure according to Anti-Hanta Virus Pool 2 “America” ELISA (IgM), Euroimmun

The samples were diluted in a 1:100 ratio with the dilution buffer containing an anti-human IgG antibody. The mixture was then vortexed and incubated for 10 minutes at room temperature. 100 µl of the calibrator, positive control, negative controls, and diluted patient samples were transferred into the respective microplates. The samples were incubated for 60 minutes at 37°C ± 1°C. The microplate was washed three times, with 300 µL of washing buffer. 100 µL of the enzyme conjugate (peroxidase-labeled anti-human IgM) was pipetted into each of the microplate wells and incubated for 30 minutes at room temperature (RT). The microplate was then washed three times as described above. 100 µl of chromogen/substrate solution was pipetted and the microplates were incubated for 15 minutes at RT. Then, 100 µl of stop solution was pipetted into each well. The color intensity was measured photometrically at a wavelength of 450 nm and a reference wavelength between 620 and 650 nm within 30 minutes of adding the stop solution.

ELISA procedure according to Anti-Hanta Virus Pool 2 “America” ELISA (IgG), Euroimmun.

The samples were diluted in a 1:100 ratio with the dilution buffer. 100 µl of the calibrator, positive control, negative controls, and diluted patient samples were transferred into the respective microplates. The incubation and washing procedures remain identical to those previously described. The sole distinction is the utilization of an enzyme conjugate (peroxidase-labeled anti-human IgG).

Calculation of the results

The extinction value of the calibrator establishes the upper limit of the reference range for non-infected individuals (cut-off), as recommended by the manufacturer. Values exceeding the specified cutoff are considered positive, while those below the cutoff are considered negative. The ratio is calculated according to the following formula:

(Extinction of the control or patient sample)/(Extinction of calibrator) = Ratio

The ratio results were interpreted as follows:

Ratio <0.8: Negative

Ratio ≥0.8 to <1.1: Borderline

Ratio ≥1.1: Positive

Reference

1. Kumar S, Stecher G, Li M, Knyaz C, Tamura K. MEGA X: Molecular Evolutionary Genetics Analysis across Computing Platforms. Mol Biol Evol. 2018;35(6):1547–9.

Appendix Table 1. List of primers used in this study

Primer name	**Primer sequence**	**Orientation**
S segment		
RM_1FO	GAAGGTATCACAATCCACGAGCAGCA	Forward
RM_1FI	AGTGGAGGTGGACCCAGATGACGT	Forward
RM_1R	CTTCATTGTGGATTGTGCTGTTGGCA	Reverse
RM_2FO	CCAACAGCACAATCCACAATGAAGGC	Forward
RM_2F1	AAGGCGGATGAAATTACACCAGGAAGA	Forward
RM_2R	AGCTGACTCTGACTCTGCTGCA	Reverse
RM_3F	CAAGCAGCAGAGTCAGAGTCAGC	Forward
RM_3RI	GGATTGTGCTAGCTGCCTGAGC	Reverse
RM_3RO	CCTTCGCATCTATCAGGGATTGTGC	Reverse
RM_4F	GCTAGCACAATCCCTGATAGATGCG	Forward
RM_4RI	CACAGTCAATGTTAGTGTTGCATAGC	Reverse
RM_4RO	GAGGTAGAAATGTGCACAGTCAATGTTAGTG	Reverse
M segment		
RM_5FO	TGCAACTTTGATCTCCATACCACCG	Forward
RM_5FI	GTGACACAACTGACACCACAGAGGC	Forward
RM_5R	CTTGAATAGCATTATCAGTACAACCTG	Reverse
RM_6F	AAACAGGTTGTACTGATAATGCTATTCAAG	Forward
RM_6RI	CTCCTGATATTGGCAGATACCCAGTCC	Reverse
RM_6RO	AATAAGCCTCACAACTTGCTCCAGG	Reverse
RM_7F	GCCCTGGAGCAAGTTGTGAGGC	Forward
RM_7RI	ACTCCAAGTGTACGGTAACATCCTCG	Reverse
RM_7RO	GCATCTACTCCTATACCGGAACACTCC	Reverse
RM_8F	CACTTGGAGTGTTCCGGTATAGGAGTAG	Forward
RM_8RI	CAGTACATCCAGTTCCAACACCAGGAC	Reverse
RM_8RO	ACCCCGCATGCAGTACATCCAGT	Reverse
RM_9FO	GCGATTGTCCTGGTGTTGGAACTGG	Forward
RM_9FI	TGGAACTGGATGTACTGCATGCGG	Forward
RM_9R	ACACTTAAACGTGCATGGAGGTGCC	Reverse
RM_10FO	GGCACCTCCATGCACGTTTAAGTG	Forward
RM_10FI	CTCCATGCACGTTTAAGTGTTGGTTCAC	Forward
RM_10R	GTATGCTCCGCAGGAACAAAAGCCT	Reverse
L segment		
RM_11F	AGTAGTAGACTCCGGGATAGAAAAGATCAG	Forward
RM_11RI	GTATCACACTGGTCTGAACTAATAGTAGAGG	Reverse
RM_11RO	ATCTCTAAACAGCCGATCCTTGTATCAC	Reverse
RM_12FO	TACTATTAGTTCAGACCAGTGTGATACAAGGA	Forward
RM_12FI	GTTCAGACCAGTGTGATACAAGGATCGG	Forward
RM_12R	ACTACCAATGTTGATACTTTCAATCATCTCACC	Reverse
RM_13F	GGTGAGATGATTGAAAGTATCAACATTGGTAG	Forward
RM_13RI	CTCCTACTGTTGAATGATCTACTGTGAGAC	Reverse
RM_13RO	TATACTACTCTAGACATAAGTGAAGGATAGACC	Reverse
RM_14FO	CTCACAGTAGATCATTCAACAGTAGGAGC	Forward
RM_14FI	GGGTCTATCCTTCACTTATGTCTAGAGTAG	Forward
RM_14R	GATGACCCTGATGCCCATCTTAAAGC	Reverse
RM_15F	AGAGAAAGCTTTAAGATGGGCATCAGG	Forward
RM_15RI	TTACATTTGCTGACACGCCACCAG	Reverse
RM_15RO	AATTCCCTTGCAGCCAATTACCTTTTAC	Reverse
RM_16FO	TTCTGGTGGCGTGTCAGCAAATGTA	Forward
RM_16FI	GTTCATCATTATTCGGCTCAGCTATTTCC	Forward
RM_16R	TGCATAGTCATTGCAGTGACAATACTAGG	Reverse
RM_17FO	AAACTGAATGATCCTAGTATTGTCACTGCA	Forward
RM_17FI	TGACTATGCAGTCACCGTTACAATTACG	Forward
RM_17RI	GATCATTTGCTTTGCACCACCTCCA	Reverse
RM_17RO	GCATTGAGCCACTCTCTTACATCCTG	Reverse
RM_18F	TATTGAAGTATGGAGGTGGTGCAAAGCA	Forward
RM_18RI	GTCACCCTTAGATCACCCTCATACAGC	Reverse
RM_18RO	TCAAGGCGTGCACAATCAAGACCA	Reverse
RM_19FO	AGAATTGATAAGATGTGGTATGAGATTCAAAACTG	Forward
RM_19FI	CCAAACACGGAGTCTTGTATTATTCAAGACA	Forward
RM_19R	GTTGAATRCCTTTCTCTCTAACAAGTAACTGTG	Reverse
RM_20FO	GGAYAGAGAAGCACAGTTACTTGTTAGAGA	Forward
RM_20FI	CACAGTTACTTGTTAGAGAGAAAGGYATTCAAC	Forward
RM_20R	TAGTAGTATGCTCCGGGAAAAGAACACT	Reverse

Complementary DNA was generated using SuperScriptIII reverse transcriptase and random hexamer primers followed by PCR using the Platinum™ Taq DNA Polymerase Kit, according to the manufacturer's instructions (https://www.thermofisher.com) and the following cycling conditions: 95°C for 3 min, followed by 45 cycles of 95°C for 30 sec, 60°C for 30 sec, and 72°C for 1 min with a final extension of 72°C 10 min. Products were purified using a GenUP™ ExoSAP kit (https://www.biotechrabbit.com/) and subsequently subjected to Sanger sequencing. O: primer used in the first round I: primer used in the second round.

Appendix Table 2. Primers and probe for qRT-PCR

Primer name	Sequence	Orientation
RIOMV F	AGGCAATCTATGATGGATTATCTG	Forward
RIOMV R	CAATCGTTCATCTCATCTATATACC	Reverse
RIOMV Probe	6FAM-TGGATGCACTTCGGAATATATATGAAACAG-IABKFQ3	Probe

The qRT-PCR reaction was carried out using the SuperScript III One-Step RT-PCR with Platinum Taq DNA Polymerase Kit (https://www.thermofisher.com) according to the manufacturer's instructions. The qRT-PCR was performed on a Roche LightCycler® 480 II with the following conditions: a reverse transcription reaction for 20 min at 55°C, followed by denaturation at 95°C for 3 min, followed by 45 cycles of 95°C for 15 sec, and 55°C for 30 sec. The viral load was calculated using a standard curve relying on a photometrically quantified in vitro-transcribed RNA control. FAM: Fluorescein amidites, IABKFQ: 3' Iowa Black® fluorescent quencher.

Appendix Table 3. Reference sequences used for phylogenetic tree inferences

Virus	Virus name abbreviation	Strain	L	M	S	Host detected	Country
Alto Paraguay virus	ALPA	A1Pa	NA	NA	DQ345762	*Holochilus chacarius*	Paraguay (PRY)
Andes virus	ANDV	Chile-9717869	AF291704	AF291703	AF291702	NA	Chile (CHI)
Araucaria virus	ARAUV	HPR/03-95	NA	NA	AY740628	NA	Brazil (BRA)
Asama virus	ASAV	N10	NA	EU929075	EU929072	*Urotrichus talpoides*	Japan (JPN)
Asikkala virus	ASIV	CZ/Beskydy/412/2010/Sm	KC880347	KC880344	KC880341	*Sorex minutus*	Czech Republic (CZE)
Bayou virus	BAYV	HV F0260003	GQ244526	L36930	L36929	*Homo sapiens*	United States of America (USA)
Bermejo virus	BMJV	Oc22531	NA	NA	AF482713	*Oligoryzomys chacoensis*	Argentina (ARG)
Black Creek Canal virus	BCCV	NA	L39951	L39950	L39949	NA	United States of America (USA)
Bowé virus	BOWV	VN1512	KC631784	KC631783	KC631782	*Crocidura douceti*	Guinea (GIN)
Bruges virus	BRUV	BE/Vieux/Genappe/TE/2013/1	KX551962	KX551961	KX551960	*Talpa europea*	Belgium (BEL)
Caño Delgadito virus	CADV	VHV-574	GQ200821	DQ284451	DQ285566	*Sigmodon alstoni*	Venezuela (VEN)
Cao Bang virus	CBV	3	EF543525	EF543526	EF543524	*Anourosorex squamipes*	Vietnam (VNM)
Carrizal virus	CARV	2/2006	NA	NA	AB620093	*Peromyscus megalops*	Mexico (MEX)
Catacamas virus	CATV	HV C1280001	NA	NA	DQ256126	*Oryzomys couesi*	Honduras (HND)
Choclo virus	CHOV	MSB96073	NA	DQ285047	DQ285046	*Oligoryzomys fulvescens*	Panama (PAN)
Convict Creek virus	CCV	Convict creek 74	NA	NA	L33816	NA	United States of America (USA)
Dabieshan virus	DABV	Yongjia-Nc-58	NA	JF796036	JF796022	*Niviventer confucianus*	China (CHN)
Dobrava-Belgrade virus	DOBV	Ano/Poroia/Afl9/1999	AJ410617	AJ410616	AJ410615	*Apodemus flavicollis*	Greece (GRC)
El Moro Canyon virus	ELMCV	RM-97	NA	U26828	U11427	*Reithrodontomys megalotis*	Mexico (MEX)
Fugong virus	FUGV	FG10	KT899703	KT899702	KT899701	*Eothenomys eleusis*	China (CHN)
Hantaan virus	HTNV	76-118	X55901	M14627	M14626	NA	Republic of Korea (KOR)
Jeju virus	JEJV	43779	HQ834697	HQ834696	HQ834695	*Crocidura shantungensis*	Republic of Korea (KOR)
Kenkeme virus	KENV	Fuyuan-Sr-326	KJ857320	KJ857337	KJ857341	*Sorex roboratus*	China (CHN)
Khabarovsk virus	KHAV	Fuyuan-Mm-217	KJ857321	KJ857338	KJ857342	*Microtus maximowiczii*	China (CHN)
Laguna Negra virus	LANV	510B	NA	AF005728	AF005727	*Calomys laucha*	Paraguay (PRY)
Lechiguanas virus	LECV	22819	NA	NA	AF482714	*Oligoryzomys flavescens*	Argentina (ARG)
Luxi virus	LUXV	LX309	HQ404253	HM756287	HM756286	*Eothenomys miletus*	China (CHN)
Maciel virus	MACV	13796	NA	NA	AF482716	*Necromys benefactus*	Argentina (ARG)

Virus	Virus name abbreviation	Strain	L	M	S	Host detected	Country
Maporal virus	MAPV	HV 97021050	EU788002	AY363179	AY267347	*Oligoryzomys fulvescens*	Venezuela (VEN)
Maripa virus	MARV	BOR	JQ611713.1	JQ611714.1	JQ611712.1	*Homo sapiens*	French Guiana (GUY)
Montano virus	MTNV	104/2006	AB620102	AB620101	AB620100	*Peromyscus beatae*	Mexico (MEX)
Necocli virus	NECV	HV O0020002	KF735065	KF494345	KF481954	*Zygodontomys brevicauda*	Colombia (COL)
New York virus	NYV	RI-I	NA	NA	UO9488	*Homo sapiens*	United States of America (USA)
Oxbow virus	OXBV	Ng1453	FJ593497	FJ539167	FJ539166	*Neurotrichus gibbsii*	United States of America (USA)
Pergamino virus	PERV	14403	NA	NA	AF482717	NA	Argentina (ARG)
Prospect Hill virus	PHV	PH-1	EF646763	X55129	Z49098	NA	United States of America (USA)
Puumala virus	PUUV	CG1820	M63194	M29979	M32750	NA	Finland (FIN)
Rio Mamore virus	RIOMV	OM-556	NA	NA	U52136	*Oligoryzomys microtis*	Bolivia (BOL)
Rio Mamore virus	RIOMV	AN683313/BRA271	NA	NA	JX443667	*Oligoryzomys microtis*	Brazil (BRA)
Rio Mamore virus	RIOMV	AN693292/BRA293	JX443697	JX443700	JX443679	*Oligoryzomys sp*	Brazil (BRA)
Rio Mamore virus	RIOMV	LH 060/2011	NA	NA	KF584259	*Homo sapiens*	Brazil (BRA)
Rio Mamore virus	RIOMV	HTN-007	FJ809772	FJ608550	FJ532244	*Oligoryzomys microtis*	Peru (PER)
Rio Segundo virus	RIOSV	RMx-Costa-1	NA	NA	U18100	*Reithrodontomys mexicanus*	Costa Rica (CRI)
Rockport virus	ROCV	MSB57412	HM015221	HM015219	M015223	*Scalopus aquaticus*	United States of America (USA)
Sangassou virus	SANGV	SA14	JQ082302	JQ082301	JQ082300	*Hylomyscus simus*	Guinea (GIN)
Seoul virus	SEOV	80-39	X56492	S47716	AY273791	*Rattus norvegicus*	Republic of Korea (KOR)
Sin Nombre virus	SNV	NM R11	L37902	L37903	L37904	*Peromyscus maniculatus*	United States of America (USA)
Tula virus	TULV	Moravia/5302v	AJ005637	Z69993	Z49915	NA	Russia (RUS)

NA: Not available.

Appendix Table 4. Reference sequences used for phylogenetic tree inferences of the Laguna Negra clade

Name	Accession no.	Virus	Country	Host
S segment				
ALPA Aipa PRY	DQ345762.1	Alto Paraguay virus	Paraguay	*Holochilus chacarius*
ANAJV AN669104 BRA 2003	HM238886.1	Anajatuba virus	Brazil	*Necromys lasiurus*
ANAJV AN690936 BRA 2003	HM238887.1	Anajatuba virus	Brazil	*Oligoryzomys fornesi*
ANAJV AN690985 BRA 2003	HM238888.1	Anajatuba virus	Brazil	*Oligoryzomys fornesi*
ANAJV H666379 BRA	HM238889.1	Anajatuba virus	Brazil	*Homo sapiens*
ANAJV H670957	HM238890.1	Anajatuba virus	Brazil	*Homo sapiens*
ANAJV H672862 BRA 2003	HM238885.1	Anajatuba virus	Brazil	*Homo sapiens*
ANAJV Of58	DQ451829.1	Anajatuba virus	NA	*Oligoryzomys fornesi*
LANV	AF005727.1	Laguna Negra virus	NA	*Calomys laucha*
LANV AN649993 BRA 2001	JQ775501.1	Laguna Negra virus	Brazil	*Callomys callidus*
LANV AN650204 BRA 2001	JQ775500.1	Laguna Negra virus	Brazil	*Callomys callidus*
LANV AN650228 BRA 2001	JQ775502.1	Laguna Negra virus	Brazil	*Callomys callidus*
LANV H650736 BRA 2001	JQ775504.1	Laguna Negra virus	Brazil	*Homo sapiens*
LANV H651686 BRA 2002	JQ775511.1	Laguna Negra virus	Brazil	*Homo sapiens*
LANV H653486 BRA 2002	JQ775514.1	Laguna Negra virus	Brazil	*Homo sapiens*
LANV H657848 BRA 2002	JQ775517.1	Laguna Negra virus	Brazil	*Homo sapiens*
LANV H660462 BRA 2002	JQ775506.1	Laguna Negra virus	Brazil	*Homo sapiens*
LANV H671696 BRA 2003	JQ775505.1	Laguna Negra virus	Brazil	*Homo sapiens*
LANV H678213 BRA 2005	JQ775513.1	Laguna Negra virus	Brazil	*Homo sapiens*
LANV H682807 BRA 2004	JQ775507.1	Laguna Negra virus	Brazil	*Homo sapiens*
LANV H695325 BRA 2005	JQ775515.1	Laguna Negra virus	Brazil	*Homo sapiens*
LANV H695689 BRA 2005	JQ775508.1	Laguna Negra virus	Brazil	*Homo sapiens*
LANV H696558 BRA 2005	JQ775512.1	Laguna Negra virus	Brazil	*Homo sapiens*
LANV H706738 BRA 2006	JQ775516.1	Laguna Negra virus	Brazil	*Homo sapiens*
LANV H710031 BRA 2006	JQ775510.1	Laguna Negra virus	Brazil	*Homo sapiens*
LANV H712518 BRA 2006	JQ775518.1	Laguna Negra virus	Brazil	*Homo sapiens*
LANV H713175 BRA 2006	JQ775509.1	Laguna Negra virus	Brazil	*Homo sapiens*
LANV LBCE 12234 BRA 2010	KP202359.1	Laguna Negra virus	Brazil	*Calomys callidus*
MARV BOR GUY 2009	JQ611712.1	Maripa virus	French Guiana	NA
MARV DOS GUY 2017	MG785209.1	Maripa virus	French Guiana	*Homo sapiens*
MARV F0102 GUY 2010	JN982967.1	Maripa virus	French Guiana	*Oligoryzomys fulvescens*
MARV GOU GUY 2008	KC876041.1	Maripa virus	French Guiana	*Homo sapiens*
MARV KES GUY 2013	MF101865.1	Maripa virus	French Guiana	*Homo sapiens*
MARV RAD GUY 2010	MF101864.1	Maripa virus	French Guiana	*Homo sapiens*
MARV VANG GUY 2016	MF101866.1	Maripa virus	French Guiana	*Homo sapiens*
RIOMM Hs85 ARG	DQ451828.1	Rio Mearim virus	NA	*Holochilus sciureus*
RIOMV 2020 PER	ERR11860590	Rio Mamore virus	Peru	*Homo sapiens*
RIOMV BRA 2004	JX443667.1	Rio Mamore virus	Brazil	*Oligoryzomys microtis*
RIOMV BRA 2005	JX443679.1	Rio Mamore virus	Brazil	*Oligoryzomys sp.*
RIOMV HTN-007 PER 1996	FJ532244.1	Rio Mamore virus	Peru	*Oligoryzomys microtis*
RIOMV LH 060/2011 BRA 2011	KF584259.1	Rio Mamore virus	Brazil	*Homo sapiens*
RIOMV OM-137 BOL	U52137.1	Rio Mamore virus	Bolivia	*Oligoryzomys microtis*
RIOMV OM-142 BOL	U52138.1	Rio Mamore virus	Bolivia	*Oligoryzomys microtis*
RIOMV OM-143 BOL	U52139.1	Rio Mamore virus	Bolivia	NA
RIOMV OM-556 BOL 1985	U52136.1	Rio Mamore virus	Bolivia	*Oligoryzomys microtis*
RIOMV OM-604 BOL	U52140.1	Rio Mamore virus	Bolivia	NA
M segment				
RIOMV HTN-007 PER 1996	FJ608550	Rio Mamore virus	Peru	*Oligoryzomys microtis*
ALPA Aipa PRY	AY515602	Alto Paraguay virus	Paraguay	*Holochilus chacarius*
LANV	NC_038506	Laguna Negra virus	NA	*Calomys laucha*
LANV AN29582 ARG 2000	JX891631	Laguna Negra virus	Argentina	*Calomys callosus*
MARV BOR GUY 2009	JQ611714.1	Maripa virus	French Guiana	*Homo sapiens*
RIOMV BRA 2004	JX443701.1	Rio Mamore virus	Brazil	*Oligoryzomys microtis*
RIOMV BRA 2005	JX443700.1	Rio Mamore virus	Brazil	*Oligoryzomys sp.*
RIOMV OM-137 BOL	U73687	Rio Mamore virus	Bolivia	*Oligoryzomys microtis*
RIOMV OM-556 BOL 1985	U73688	Rio Mamore virus	Bolivia	*Oligoryzomys microtis*

NA: Not available.

Appendix Table 5. Amino acid distance matrix for complete nucleoprotein sequences using a p-distance substitution model. The final data set contained 433 positions for the nucleoprotein, analyses were conducted in MEGA X (*1*)

Virus	RIOMV	MARV	LANV	ANDV	SNV	DOBV	HTNV	PUUV	SAAV	SEOV
RIOMV PER 1996 FJ532244										
MARV GUY KC876041	0.0280									
LANV PRY NC_038505	0.0724	0.0724								
ANDV CHI NC_003466	0.0888	0.0864	0.0958							
SNV USA L37904	0.1542	0.1495	0.1472	0.1402						
DOBV GRC AJ410615	0.3668	0.3645	0.3598	0.3528	0.3715					
HTNV KOR M14626	0.3528	0.3528	0.3551	0.3481	0.3715	0.1702				
PUUV FIN NC_005224	0.2757	0.2757	0.2757	0.2710	0.2921	0.3846	0.3893			
SAAV DEN AJ616854	0.3738	0.3715	0.3668	0.3598	0.3738	0.0140	0.1655	0.3846		
SEOV KOR AY273791	0.3621	0.3551	0.3621	0.3528	0.3762	0.1841	0.1678	0.3753	0.1772	

Appendix Table 6. Amino acid distance matrix for partial nucleoprotein sequences using a p-distance model. The final data set contained 276 positions for the nucleoprotein, analyses were conducted in MEGA X (*1*)

Virus	RIOMV 2020	RIOMV 1996	MAPV	LANV	ANDV	SNV
RIOMV 2020						
RIOMV PER 1996 FJ532244	0.0000					
MARV GUY KC876041	0.0291	0.0291				
LANV PRY NC_038505	0.0436	0.0436	0.0509			
ANDV CHI NC_003466	0.0655	0.0655	0.0691	0.0655		
SNV USA L37904	0.1200	0.1200	0.1164	0.1055	0.1055	

Appendix Table 7. Amino acid distance matrix for glycoprotein sequences using a p-distance model. The final data set contained 1139 positions for the glycoprotein, analyses were conducted in MEGA X (*1*). Saaremaa virus was not included in the study due to the absence of a complete M sequence.

Virus	RIOMV	MARV	LANV	ANDV	SNV	DOBV	HTNV	PUUV	SEOV
RIOMV PER 1996 FJ608550									
MARV GUY JQ611714	0.0378								
LANV PRY AF005728	0.0879	0.0914							
ANDV CHI NC_003467	0.1230	0.1221	0.1353						
SNV USA L37903	0.2250	0.2293	0.2320	0.2206					
DOBV GRC AJ410616	0.4527	0.4500	0.4589	0.4580	0.4589				
HTNV KOR M14627	0.4466	0.4457	0.4537	0.4501	0.4501	0.2259			
PUUV FIN M29979	0.3369	0.3404	0.3456	0.3369	0.3275	0.4660	0.4625		
SEOV KOR NC_005237	0.4571	0.4571	0.4598	0.4571	0.4686	0.2277	0.2286	0.4633	

Appendix Table 8. Comparison of IgG ELISA reactivity in complementary testing

		Ecozone		
Type of ELISA/ Group		Lambayeque	Lima	Loreto
CHIKV	Hantavirus IgG ELISA-positive samples	0%	0%	25% (n=2)
	Control group	0%	0%	7.7% (n=1)
	Total	0%	0%	14.3% (n=3),
MAYV	Hantavirus IgG ELISA-positive samples	0%	0%	37.5% (n=3)
	Control group	0%	15.4% (n=2)	7.7% (n=1)
	Total*	0%	11.8% (n=2)	19.0% (n=4)
OROV	Hantavirus IgG ELISA-positive samples	0%	0%	12.5% (n=1)
	Control group	0%	0%	38.5% (n=5)
	Total	0%	0%	28.6% (n=6)
Plasmodium	Hantavirus IgG ELISA-positive samples	62.5% (n=5)	75% (n=3)	37.5% (n=3)
	Control group	8.3% (n=1)	30.8% (n=4)	7.7% (n=1)
	Total†	30% (n=6)	41.2% (n=7)	20% (n=4)
SARS-CoV-2	Hantavirus IgG ELISA-positive samples	0%	0%	12.5% (n=1)
	Control group	8.3% (n=1)	0%	7.7% (n=1)
	Total‡	5% (n=1)	0%	10% (n=2)

CHIKV: Chikungunya virus, MAYV: Mayaro virus, OROV: Oropouche virus, SARS-CoV-2 S: Severe acute respiratory syndrome coronavirus 2. The results excluded the two sera that tested positive for cytomegalovirus. Hantavirus IgG ELISA-positive samples: Lambayeque n=8, Lima n=4, and Loreto n=8. Control group: Lambayeque n=12, Lima n=13 and Loreto n=13. Cells with zero-cell counts were excluded from the statistical ..analysis.
*Total samples from Lima and Loreto were compared by a Fisher`s exact test: *p*=0.7;
†: Total samples from Lambayeque, Lima, and Loreto were compared by a Chi-Square test: X^2=2.2, *p*=0.33; ‡: Total samples from Lambayeque, and Loreto were compared by a Fisher`s exact test: *p*=0.2.

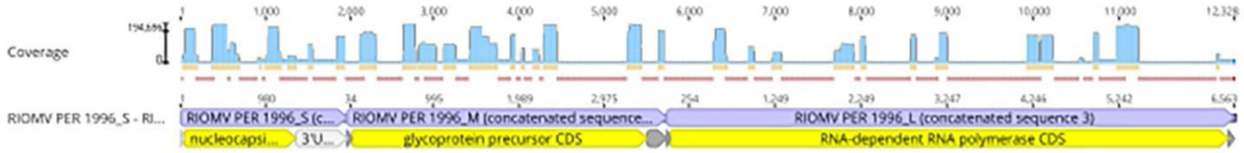

Appendix Figure 1. Coverage of Rio Mamore 2020 (RIOMV 2020) segments compared to Rio Mamore 1996 (RIOMV 1996) strain. The partial sequences obtained from high-throughput and Sanger sequencing are shown in orange. The alignment was constructed using Geneious (2023.2.1). The nucleotide positions are indicated by segment.

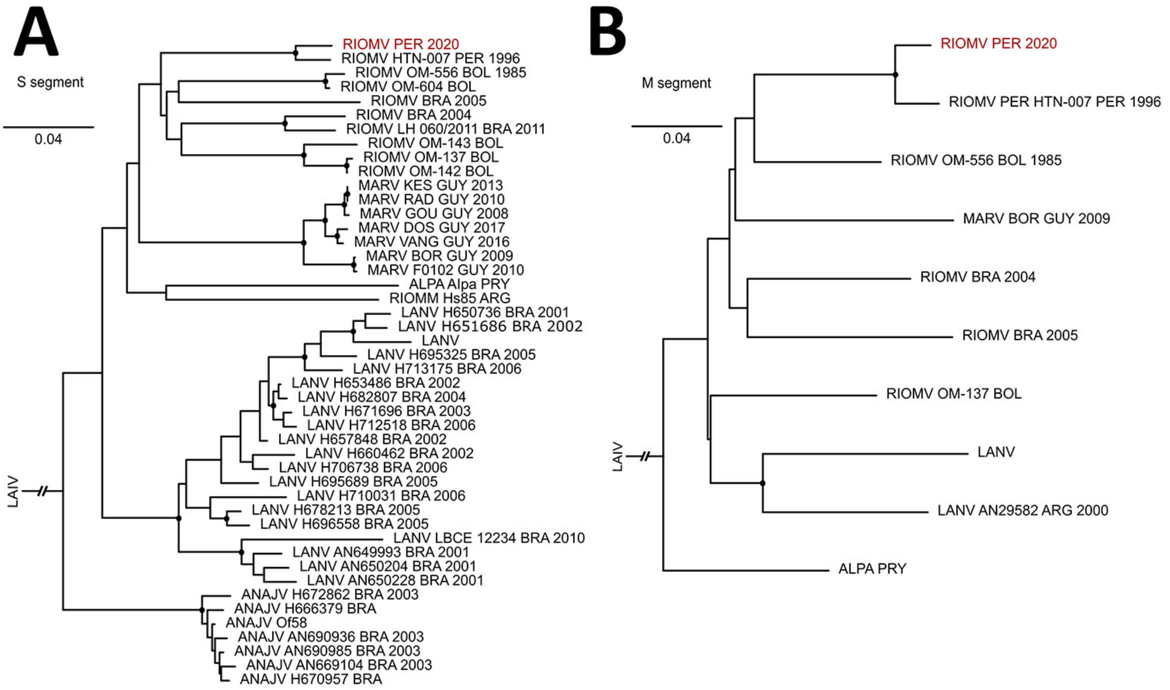

Appendix Figure 2. Phylogenetic relationships between the Rio Mamore virus from Peru (RIOMV PER 2020, shown in red) and *Orthohantavirus mamorense* species. A) Partial sequence of the S segment (276 nt). B) Partial sequence of the M segment (150 nt). Phylogenetic trees were constructed using a Neighbor-joining (NJ) method with 1000 bootstraps and p-distance. The percentage of replicate trees in which the associated taxa clustered ≥0.80 in the bootstrap test is marked as a black dot. The location, year, and strain are indicated when available. Due to the low overlap of partial genome sequences available in GenBank, resulting in a small dataset of limited phylogenetic information, simplistic NJ trees were used instated of probabilistic methods as used for larger genomic sequences.

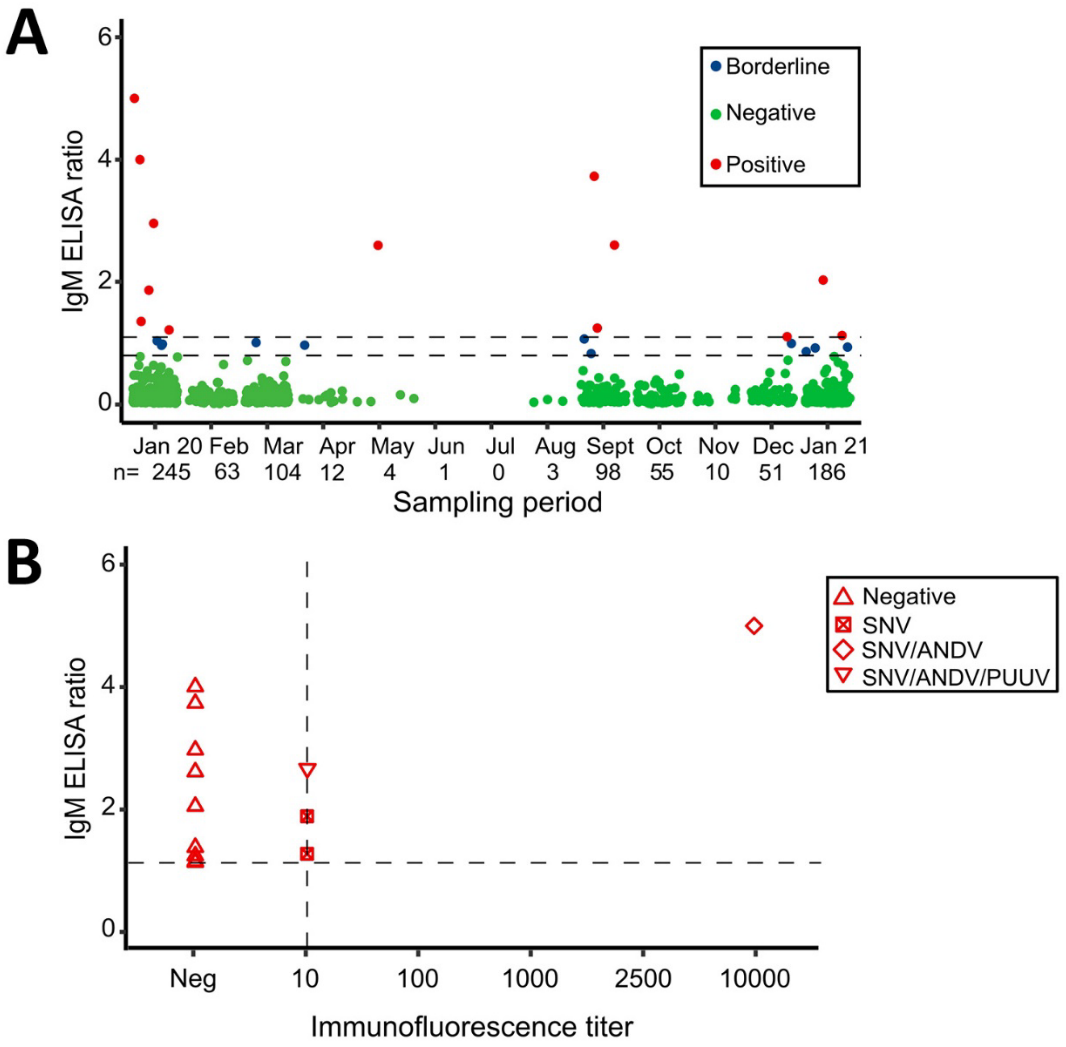

Appendix Figure 3. Hantavirus incidence in the Peruvian amazon, 2020–2021. A) Andes virus (ANDV) and Sin Nombre virus (SNV) IgM ELISA reactivities in Loreto, Peru, between January 2020 and January 2021, single study datum is indicated as a dot. B) ANDV and SNV positive serum samples in IgM ELISA compared to IgM immunofluorescence assay (IFA) titers.

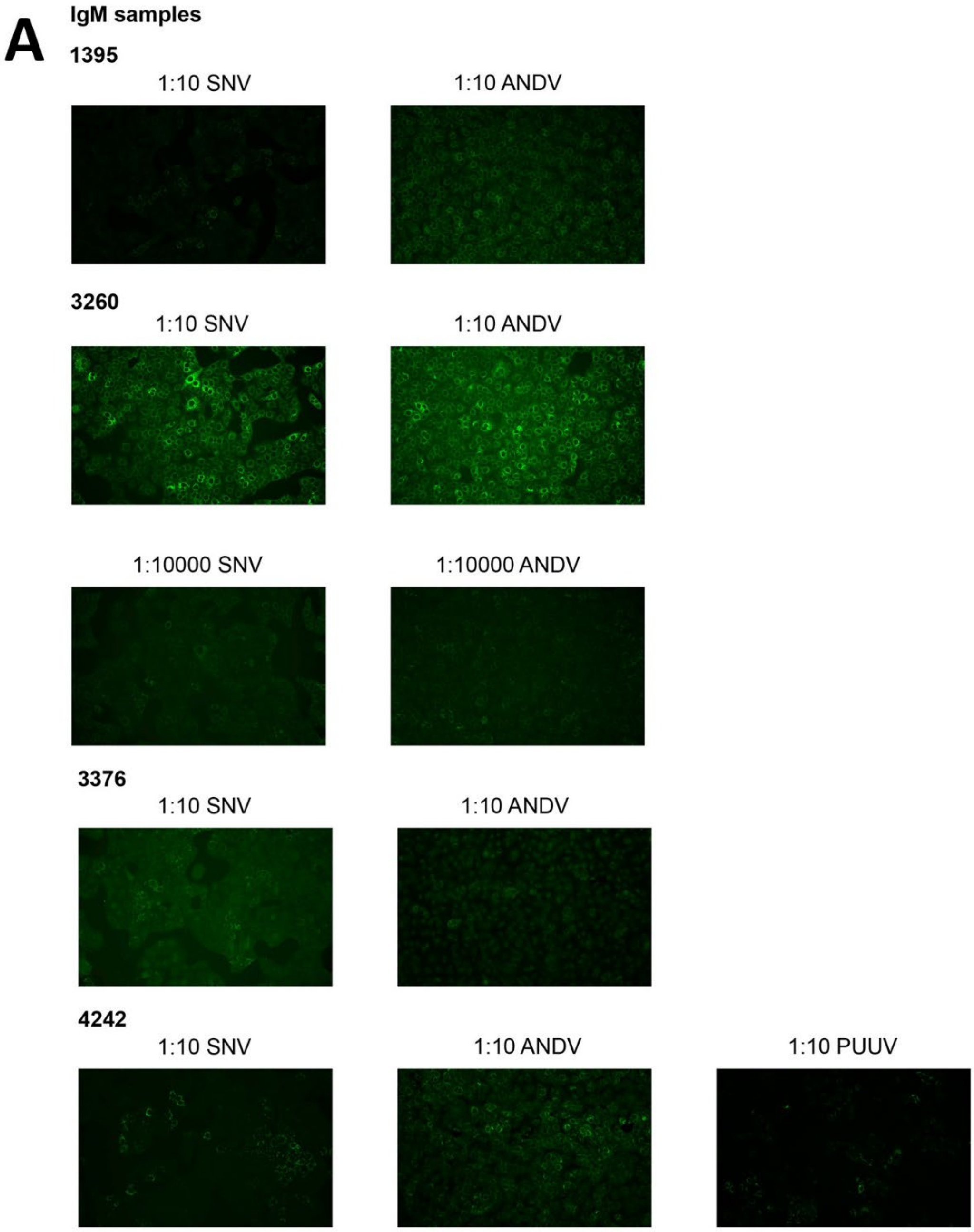
A
IgM samples
1395
1:10 SNV
1:10 ANDV
3260
1:10 SNV
1:10 ANDV
1:10000 SNV
1:10000 ANDV
3376
1:10 SNV
1:10 ANDV
4242
1:10 SNV
1:10 ANDV
1:10 PUUV

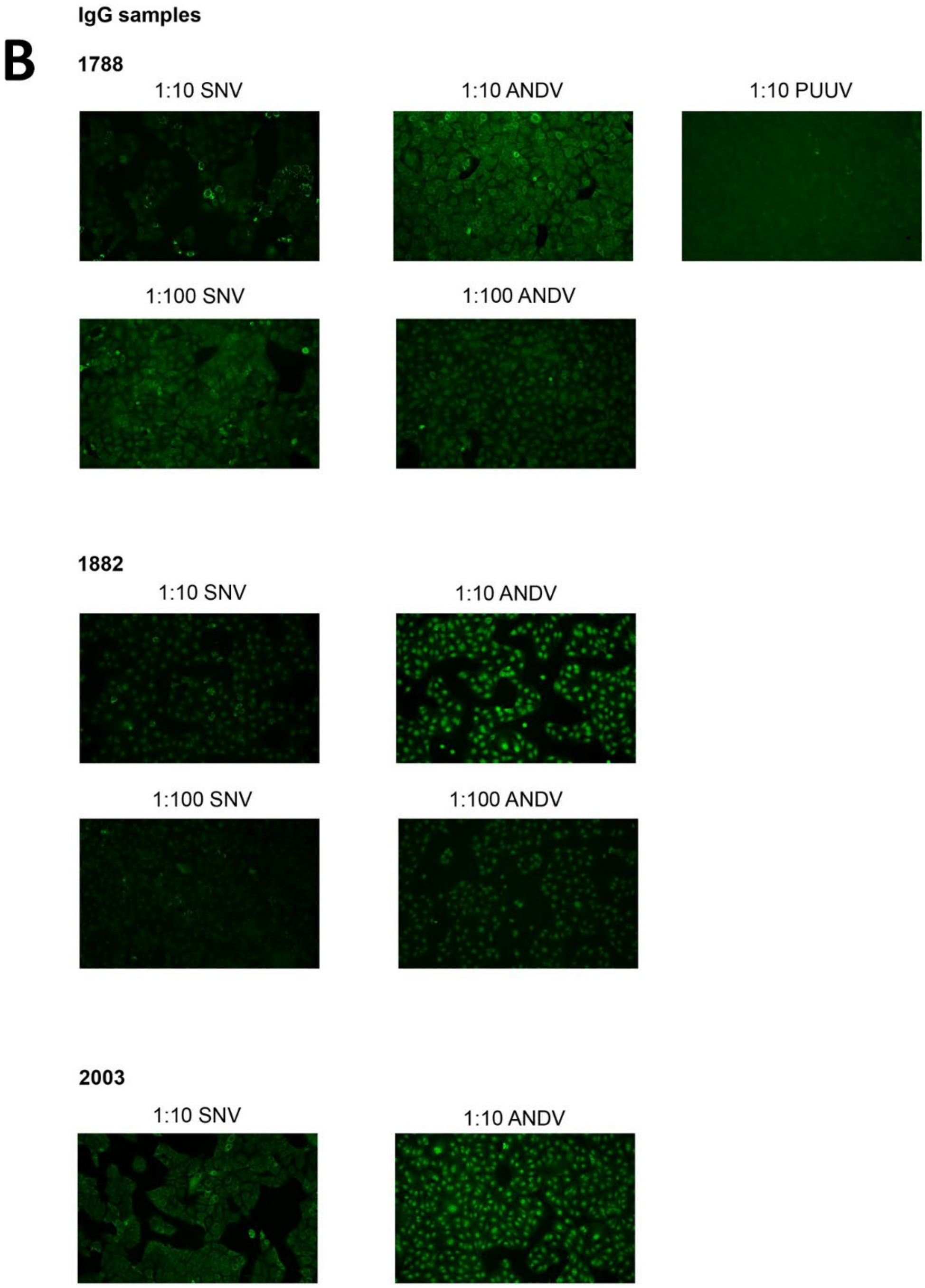
IgG samples
B
1788
1:10 SNV
1:10 ANDV
1:10 PUUV
1:100 SNV
1:100 ANDV
1882
1:10 SNV
1:10 ANDV
1:100 SNV
1:100 ANDV
2003
1:10 SNV
1:10 ANDV

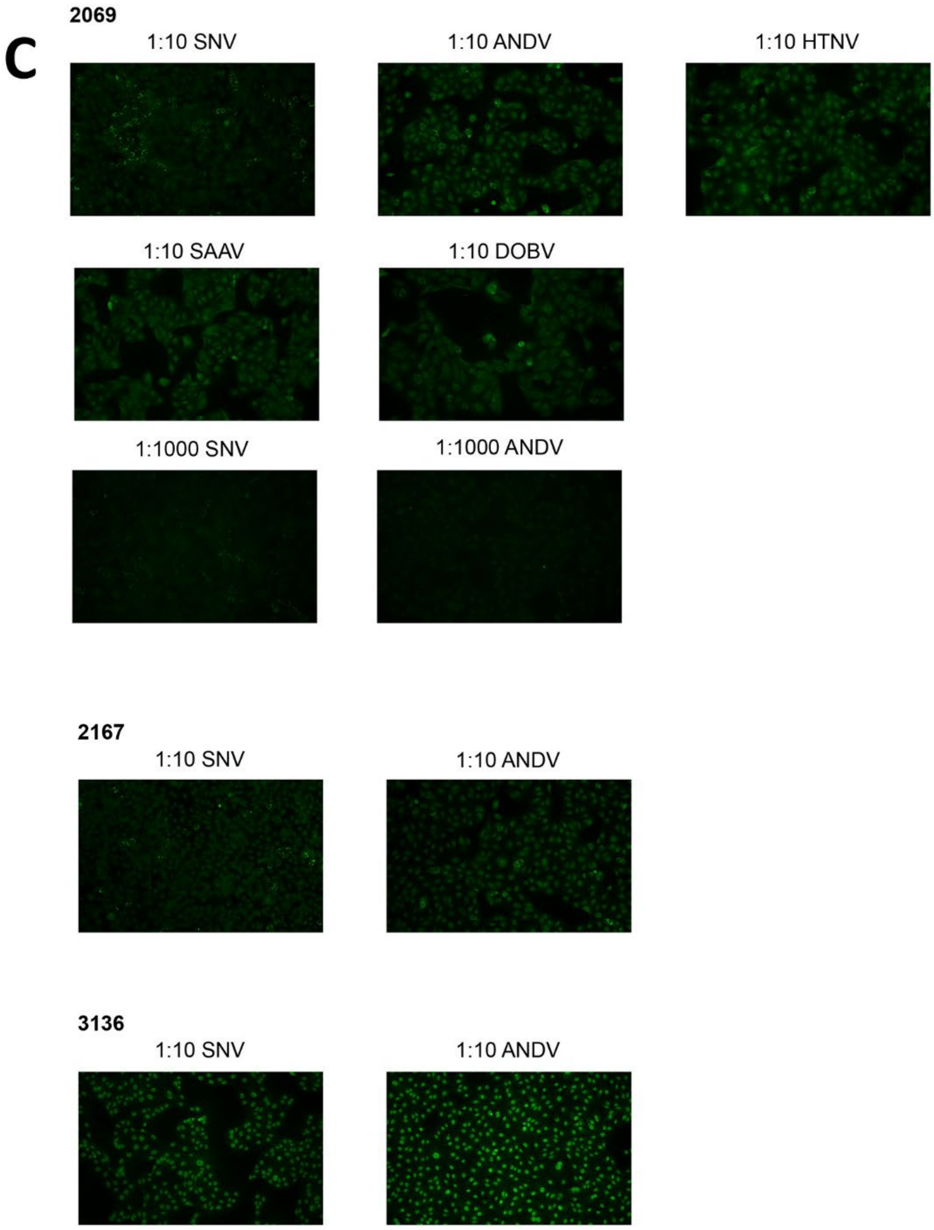
C
2069
1:10 SNV
1:10 ANDV
1:10 HTNV
1:10 SAAV
1:10 DOBV
1:1000 SNV
1:1000 ANDV
2167
1:10 SNV
1:10 ANDV
3136
1:10 SNV
1:10 ANDV

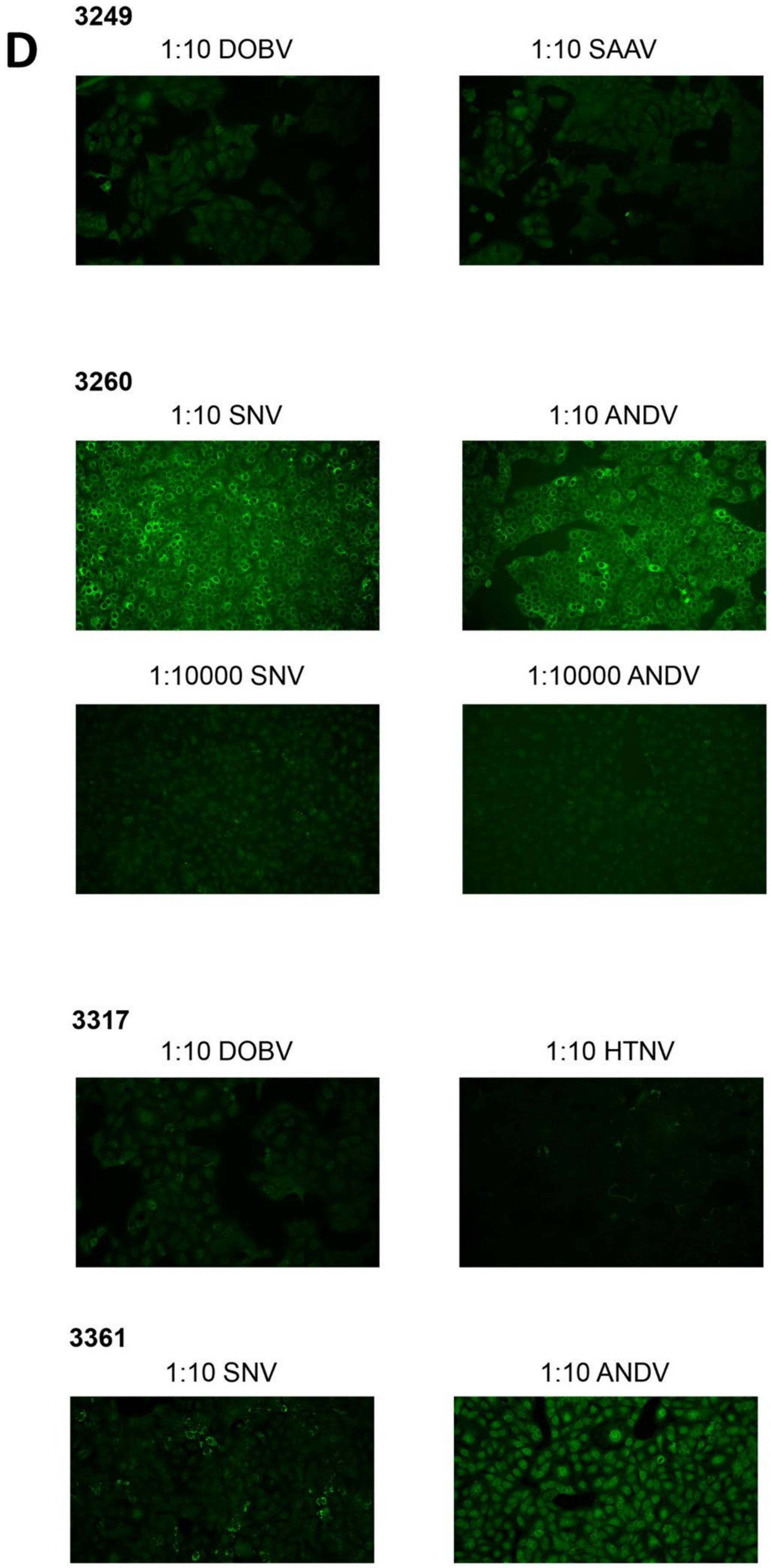
D
3249
1:10 DOBV
1:10 SAAV
3260
1:10 SNV
1:10 ANDV
1:10000 SNV
1:10000 ANDV
3317
1:10 DOBV
1:10 HTNV
3361
1:10 SNV
1:10 ANDV

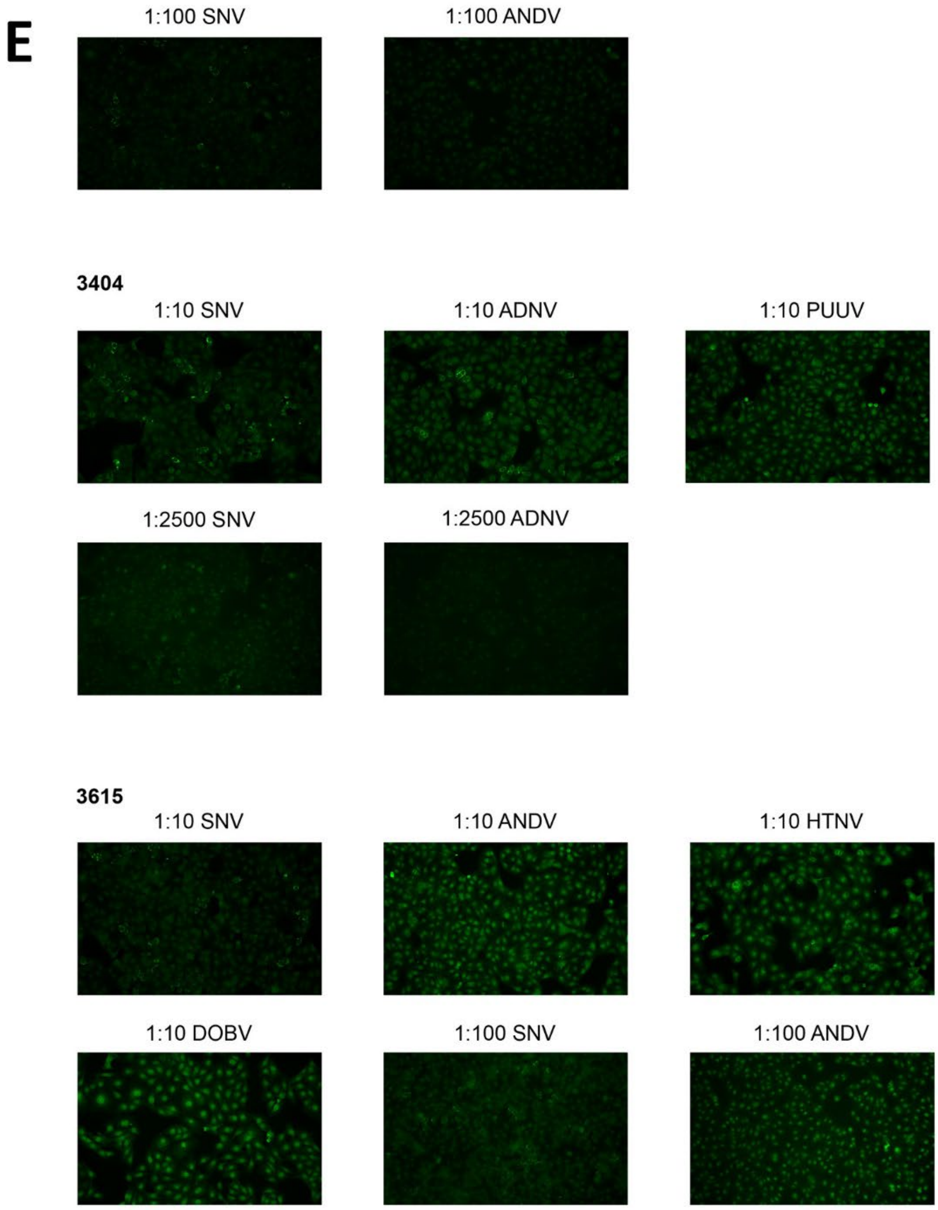
E
1:100 SNV
1:100 ANDV
3404
1:10 SNV
1:10 ADNV
1:10 PUUV
1:2500 SNV
1:2500 ADNV
3615
1:10 SNV
1:10 ANDV
1:10 HTNV
1:10 DOBV
1:100 SNV
1:100 ANDV

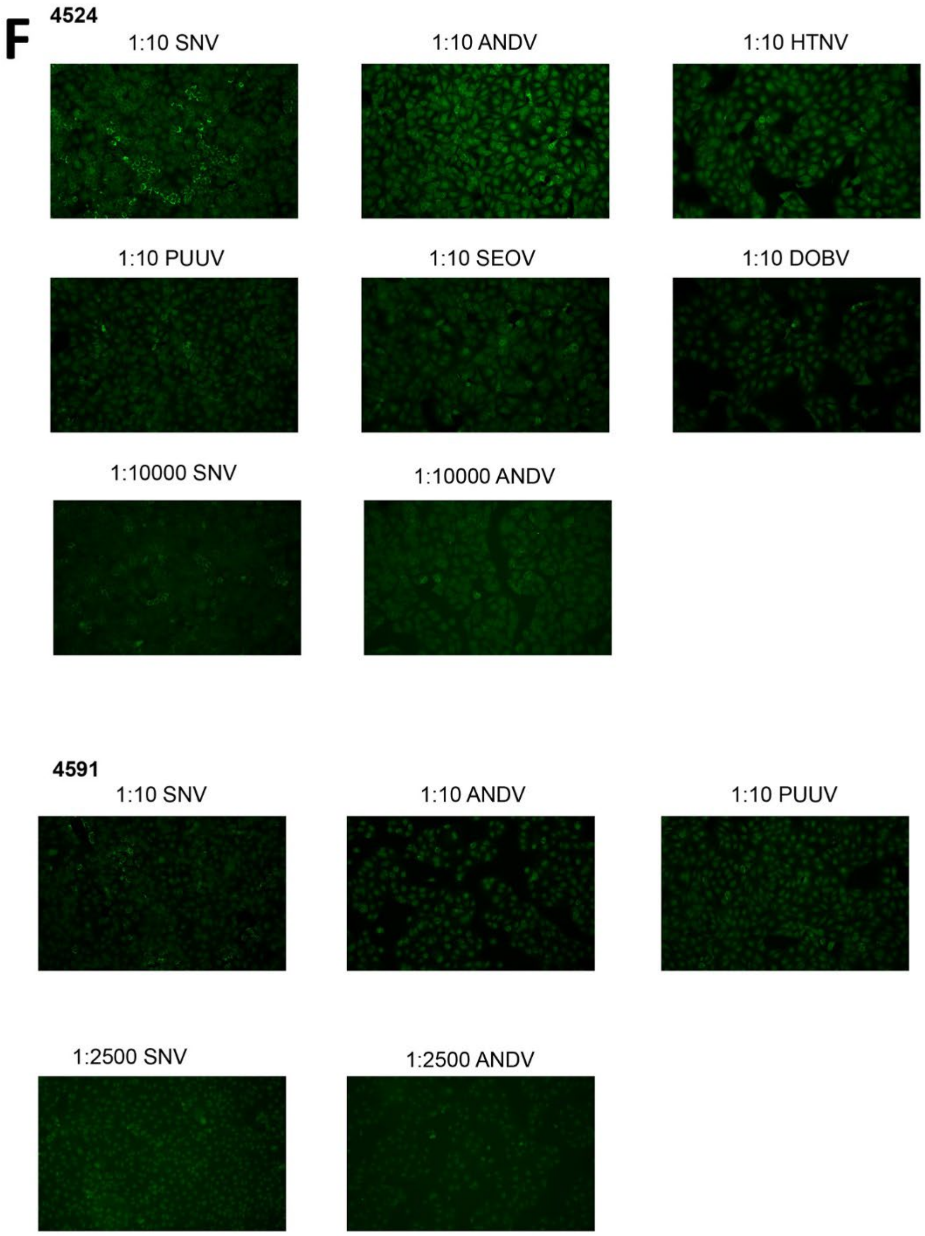
F
4524
1:10 SNV
1:10 ANDV
1:10 HTNV
1:10 PUUV
1:10 SEOV
1:10 DOBV
1:10000 SNV
1:10000 ANDV
4591
1:10 SNV
1:10 ANDV
1:10 PUUV
1:2500 SNV
1:2500 ANDV

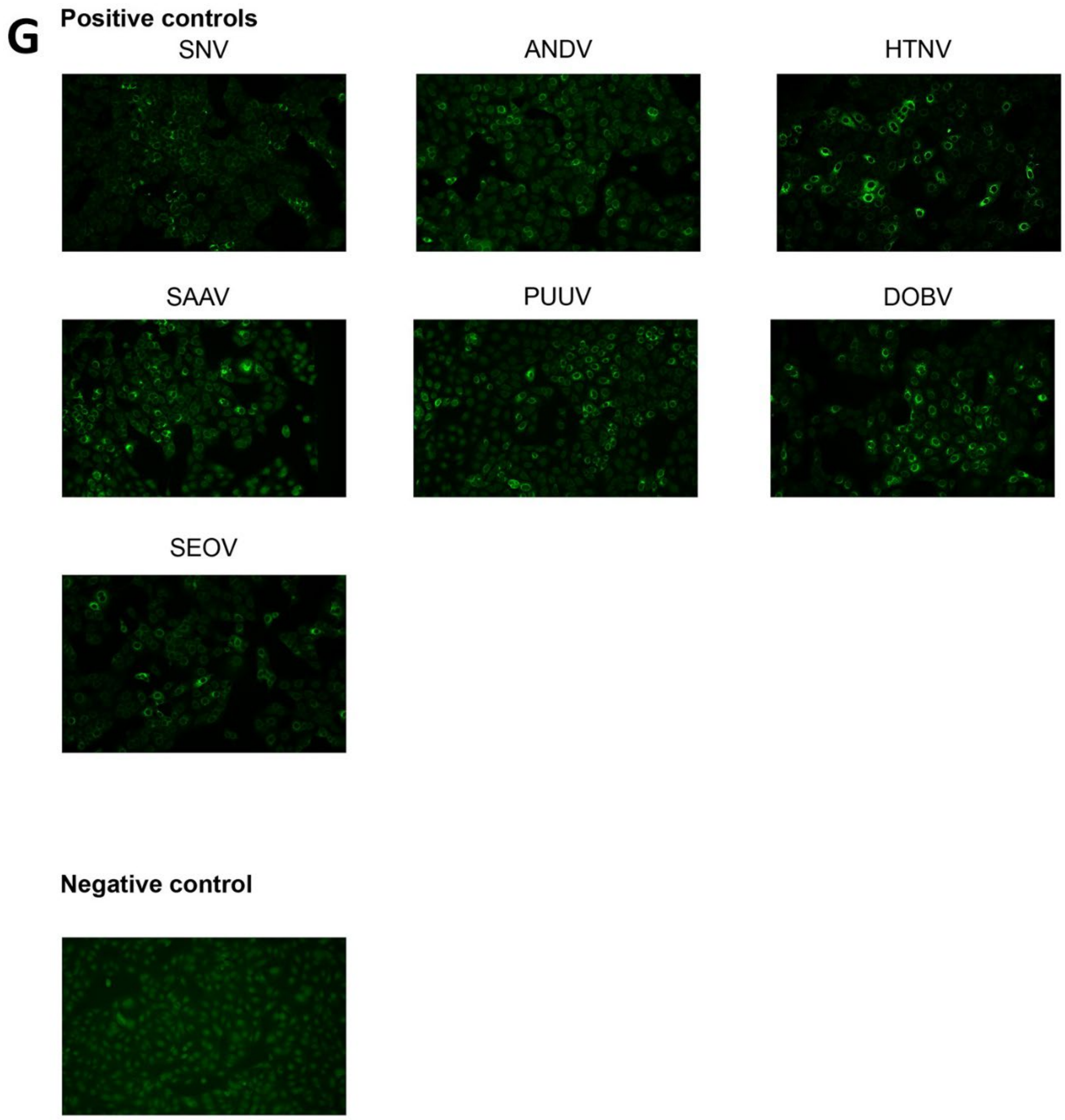

Appendix Figure 4. End-point titration using immunofluorescence assay (IFA) for IgM and IgG antibodies against Sin Nombre virus (SNV), Andes virus (ANDV), Seoul virus (SEOV), Hantaan (HTNV), Puumala (PUUV), Dobrava (DOBV), and Saaremaa (SAAV) hantaviruses. Samples IDs are indicated to the upper left of panels. All images were taken at 200 magnification.

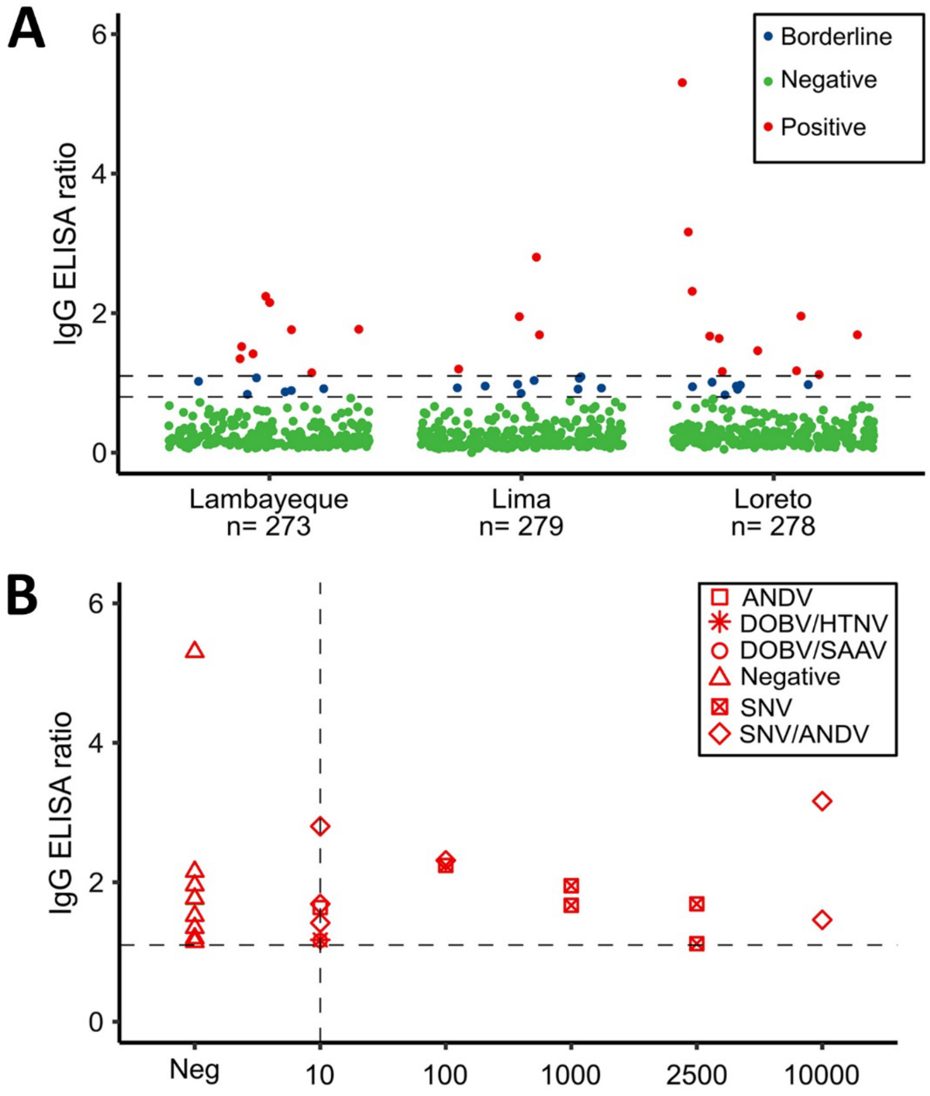

Appendix Figure 5. Hantavirus seroepidemiology in Peru. A) ANDV and SNV IgG reactivities in 3 departments of Peru, between January 2020 and January 2021. B) ANDV- and SNV-positive serum samples in IgG ELISA compared to IgG IFA titers. DOBV, Dobrava virus, HTNV, Hantaan virus, SAAV, Saaremaa virus.
